# MerTK is a mediator of alpha-synuclein fibril uptake by human microglia

**DOI:** 10.1093/brain/awad298

**Published:** 2023-09-06

**Authors:** Marie-France Dorion, Moein Yaqubi, Konstantin Senkevich, Nicholas W Kieran, Adam MacDonald, Carol X Q Chen, Wen Luo, Amber Wallis, Irina Shlaifer, Jeffery A Hall, Roy W R Dudley, Ian A Glass, Birth Defects Research Laboratory, Jo Anne Stratton, Edward A Fon, Tim Bartels, Jack P Antel, Ziv Gan-or, Thomas M Durcan, Luke M Healy

**Affiliations:** Early Drug Discovery Unit, Montreal Neurological Institute-Hospital, McGill University, Montreal H3A 2B4, Canada; Neuroimmunology Unit, Montreal Neurological Institute-Hospital, McGill University, Montreal H3A 2B4, Canada; Department of Neurology and Neurosurgery, Montreal Neurological Institute-Hospital, McGill University, Montreal H3A 2B4, Canada; Neuroimmunology Unit, Montreal Neurological Institute-Hospital, McGill University, Montreal H3A 2B4, Canada; Department of Neurology and Neurosurgery, Montreal Neurological Institute-Hospital, McGill University, Montreal H3A 2B4, Canada; McGill Parkinson Program and Neurodegenerative Diseases Group, Montreal Neurological Institute-Hospital, McGill University, Montreal H3A 2B4, Canada; Department of Human Genetics, McGill University, Montreal H3A 0C7, Canada; Neuroimmunology Unit, Montreal Neurological Institute-Hospital, McGill University, Montreal H3A 2B4, Canada; Neuroimmunology Unit, Montreal Neurological Institute-Hospital, McGill University, Montreal H3A 2B4, Canada; Department of Neurology and Neurosurgery, Montreal Neurological Institute-Hospital, McGill University, Montreal H3A 2B4, Canada; Early Drug Discovery Unit, Montreal Neurological Institute-Hospital, McGill University, Montreal H3A 2B4, Canada; Department of Neurology and Neurosurgery, Montreal Neurological Institute-Hospital, McGill University, Montreal H3A 2B4, Canada; Early Drug Discovery Unit, Montreal Neurological Institute-Hospital, McGill University, Montreal H3A 2B4, Canada; Department of Neurology and Neurosurgery, Montreal Neurological Institute-Hospital, McGill University, Montreal H3A 2B4, Canada; UK Dementia Research Institute, University College London, London WC1E 6BT, UK; Early Drug Discovery Unit, Montreal Neurological Institute-Hospital, McGill University, Montreal H3A 2B4, Canada; Department of Neurology and Neurosurgery, Montreal Neurological Institute-Hospital, McGill University, Montreal H3A 2B4, Canada; Department of Neurology and Neurosurgery, Montreal Neurological Institute-Hospital, McGill University, Montreal H3A 2B4, Canada; Department of Pediatric Surgery, Division of Neurosurgery, Montreal Children's Hospital, McGill University Health Centers, Montreal H4A 3J1, Canada; Department of Pediatrics, University of Washington, Seattle, WA 98195, USA; Department of Pediatrics, University of Washington, Seattle, WA 98195, USA; Neuroimmunology Unit, Montreal Neurological Institute-Hospital, McGill University, Montreal H3A 2B4, Canada; Department of Neurology and Neurosurgery, Montreal Neurological Institute-Hospital, McGill University, Montreal H3A 2B4, Canada; Early Drug Discovery Unit, Montreal Neurological Institute-Hospital, McGill University, Montreal H3A 2B4, Canada; Department of Neurology and Neurosurgery, Montreal Neurological Institute-Hospital, McGill University, Montreal H3A 2B4, Canada; McGill Parkinson Program and Neurodegenerative Diseases Group, Montreal Neurological Institute-Hospital, McGill University, Montreal H3A 2B4, Canada; UK Dementia Research Institute, University College London, London WC1E 6BT, UK; Neuroimmunology Unit, Montreal Neurological Institute-Hospital, McGill University, Montreal H3A 2B4, Canada; Department of Neurology and Neurosurgery, Montreal Neurological Institute-Hospital, McGill University, Montreal H3A 2B4, Canada; McGill Parkinson Program and Neurodegenerative Diseases Group, Montreal Neurological Institute-Hospital, McGill University, Montreal H3A 2B4, Canada; Department of Human Genetics, McGill University, Montreal H3A 0C7, Canada; Early Drug Discovery Unit, Montreal Neurological Institute-Hospital, McGill University, Montreal H3A 2B4, Canada; Department of Neurology and Neurosurgery, Montreal Neurological Institute-Hospital, McGill University, Montreal H3A 2B4, Canada; Neuroimmunology Unit, Montreal Neurological Institute-Hospital, McGill University, Montreal H3A 2B4, Canada; Department of Neurology and Neurosurgery, Montreal Neurological Institute-Hospital, McGill University, Montreal H3A 2B4, Canada

**Keywords:** microglia, MerTK, alpha-synuclein, phagocytosis, Parkinson’s disease

## Abstract

Mer tyrosine kinase (MerTK) is a receptor tyrosine kinase that mediates non-inflammatory, homeostatic phagocytosis of diverse types of cellular debris. Highly expressed on the surface of microglial cells, MerTK is of importance in brain development, homeostasis, plasticity and disease. Yet, involvement of this receptor in the clearance of protein aggregates that accumulate with ageing and in neurodegenerative diseases has yet to be defined. The current study explored the function of MerTK in the microglial uptake of alpha-synuclein fibrils which play a causative role in the pathobiology of synucleinopathies.

Using human primary and induced pluripotent stem cell-derived microglia, the MerTK-dependence of alpha-synuclein fibril internalization was investigated *in vitro*. Relevance of this pathway in synucleinopathies was assessed through burden analysis of *MERTK* variants and analysis of MerTK expression in patient-derived cells and tissues.

Pharmacological inhibition of MerTK and siRNA-mediated *MERTK* knockdown both caused a decreased rate of alpha-synuclein fibril internalization by human microglia. Consistent with the non-inflammatory nature of MerTK-mediated phagocytosis, alpha-synuclein fibril internalization was not observed to induce secretion of pro-inflammatory cytokines such as IL-6 or TNF, and downmodulated IL-1β secretion from microglia. Burden analysis in two independent patient cohorts revealed a significant association between rare functionally deleterious *MERTK* variants and Parkinson’s disease in one of the cohorts (*P* = 0.002). Despite a small upregulation in *MERTK* mRNA expression in nigral microglia from Parkinson’s disease/Lewy body dementia patients compared to those from non-neurological control donors in a single-nuclei RNA-sequencing dataset (*P* = 5.08 × 10^−21^), no significant upregulation in MerTK protein expression was observed in human cortex and substantia nigra lysates from Lewy body dementia patients compared to controls.

Taken together, our findings define a novel role for MerTK in mediating the uptake of alpha-synuclein fibrils by human microglia, with possible involvement in limiting alpha-synuclein spread in synucleinopathies such as Parkinson’s disease. Upregulation of this pathway in synucleinopathies could have therapeutic values in enhancing alpha-synuclein fibril clearance in the brain.

## Teaser

Dorion *et al.* show that the protein MerTK is involved in the internalization of alpha-synuclein fibrils by microglia. Upregulating this process could have therapeutic potential in disorders such as Parkinson’s disease by enhancing the clearance of alpha-synuclein aggregates from the brain.

## Introduction

Synucleinopathies are neurodegenerative disorders characterized by the intracellular accumulation of aggregated alpha-synuclein (α-syn) protein. Broadly, they are classified into multiple system atrophy and Lewy body diseases that include Parkinson’s disease and Lewy body dementia. The neuronal accumulation of α-syn in the form of Lewy bodies and its role in neurodegenerative processes have been extensively studied. Duplication or triplication of the genetic locus encoding α-syn (*SNCA*) have been identified to cause autosomal dominant Parkinson’s disease,^1^ implying that accumulation of excess α-syn is sufficient to trigger the disease process. Current evidence supports the notion that the initiation of α-syn aggregation, either in the CNS or peripheral tissues, can then propagate in a prion-like manner into the brain, where it induces neuronal dysfunction and degeneration.^2-7^ Accordingly, injection of recombinant α-syn preformed fibrils (PFFs) into mice leads to the development of Lewy body-like pathologies, neuronal loss and behavioural deficits.^8^*In vitro,* seeding of neurons with α-syn PFFs also results in the formation of Lewy body-like inclusions and dysregulation of cellular processes.^9,10^

Strategies to prevent cell-to-cell spread of α-syn have been proposed as potential therapies to prevent neurodegeneration in synucleinopathies.^11^ Mechanisms involved in α-syn endocytosis are being actively explored, with differential internalization mechanisms observed across brain cell types. For instance, heparan sulfate proteoglycans have been identified as mediators of α-syn PFF internalization by neurons but not microglia.^12^ Microglia, as professional phagocytes residing in the brain, can internalize and degrade α-syn fibrils at a higher rate than other cells,^13-15^ making them a particularly promising cell type that can be targeted in the development of novel therapeutics to promote α-syn clearance. The majority of microglia studies investigating α-syn internalization processes to date have been carried out using murine microglia or the murine microglia-like cell line BV2 and have contributed to the identification of a variety of cell surface proteins that interact with human α-syn, including toll-like receptors (TLRs) and cluster of differentiation 36.^16-20^ Since human and murine microglia are inherently different in their genome, transcriptome and aspects of their function,^21-24^ it is unclear whether the same receptors and mechanisms participate in the uptake of α-syn by human microglia.

In a previous study comparing the impact of culture media formulation on microglia phagocytic activities, we observed a correlation between α-syn fibril and myelin debris uptake activities^25^ suggestive of a common mechanism of internalization for these substrates. Both α-syn fibril and myelin debris uptake activities also correlated with the expression level of Mer tyrosine kinase (MerTK), a microglial surface receptor kinase with known roles in myelin phagocytosis.^26^

TYRO3, AXL and MerTK constitute the ‘TAM’ family of homologous receptor tyrosine kinases with pleiotropic functions. AXL and MerTK are particularly known for their roles in the phagocytosis of apoptotic cells,^27^ myelin debris,^26^ synapses^28^ and photoreceptor outer segments.^29,30^ Tethering of phagocytic targets to TAM receptors occurs through the binding of secreted bridging molecules—growth arrest-specific 6 (GAS6) and protein S1 (PROS1)—with high affinity for phosphatidylserine moieties exposed on the membranes of dead cells.^31^ Binding of GAS6/PROS1 to TAM receptors triggers activation of the cytoplasmic kinase domain, autophosphorylation and downstream signalling that induces the cytoskeletal remodelling required for phagocytosis.^31^ This process is immunologically silent and suppresses the inflammatory activation of myeloid cells during homeostatic phagocytosis.^31,32^

High expression of MerTK, as well as its activating ligands GAS6 and PROS1, is a characteristic feature of microglia that distinguishes them from other myeloid populations.^33^ The present study aimed to investigate the role of MerTK in α-syn fibril uptake by human primary and induced pluripotent stem cell-derived microglia (iMGL). The relevance of this process in synucleinopathies was evaluated through rare variant burden analysis of *MERTK* in Parkinson’s disease and assessment of MerTK expression in patient-derived brain materials. Taken together, we show that α-syn fibril uptake by human microglia is largely MerTK-dependent. Lack of MerTK upregulation accompanying α-syn accumulation in the human brain and genetic associations between rare *MERTK* variants and Parkinson’s disease suggest MerTK as a potential therapeutic target to enhance α-syn fibril clearance by microglia in synucleinopathies.

## Materials and methods

### Ethical approval

Use of all human materials was approved by the McGill University Health Centre Research Ethics Board under project no. 1989-178 for human brain tissues and 2019-5374 for induced pluripotent stem cells (iPSCs).

### Microglia isolation and culture

Cortical tissues from 2- to 68-year-old female and male epilepsy patients were obtained from the Montreal Neurological Institute, Montreal, Canada (adult donors), and the Montreal Children’s Hospital, Montreal, Canada (paediatric donors), with written consent and under approval from local ethics boards. Tissue samples were aspirated into ultrasonic surgical aspirator bags (CUSA^®^) from the surgical ‘corridor’ away from the site of suspected epileptic foci. Materials were comprised predominantly of white matter tissue. Focal or diffused pathology were excluded as much as possible during the operative procedure and retrospective histological analyses. Samples were excluded from the study when brain tumours were discovered to be associated with epileptic seizures. Second trimester fetal cerebrum tissues were obtained from Centre Hospitalier Universitaire Sainte-Justine, Montreal, Canada, or from the Birth Defects Research Laboratory, University of Washington, Seattle, USA, with maternal written consent and under approval from local ethics boards. Demographic information about the donors is included in Supplementary Table 1. Isolation of glial cells was carried out as previously described^34^ through mechanical and chemical digestion, followed by Percoll^®^ (Sigma-Aldrich) gradient centrifugation (postnatal tissue) or not (fetal tissue). Microglia were further purified by taking advantage of the differential adhesive properties of the glial cells. This resulted in a culture purity of ∼97% as assessed by PU.1 immunostaining (Supplementary Fig. 1A and B). *Ex vivo* human postnatal microglia (hMGL) were prepared by magnetic activated bead sorting of CD11b+ cells immediately after tissue processing as previously described.^25^ HMGL were maintained *in vitro* in microglia growth medium composed of Dulbecco’s modified Eagle’s medium (DMEM)/F12 (Thermo Fisher Scientific), 1% GlutaMAX^TM^ (Thermo Fisher Scientific), 1% non-essential amino acids (Thermo Fisher Scientific), 1% penicillin/streptomycin (P/S; Thermo Fisher Scientific), 2× insulin-transferrin-selenium (Thermo Fisher Scientific), 2× B27 (Thermo Fisher Scientific), 0.5× N2 (Thermo Fisher Scientific), 400 μM monothioglycerol (Sigma Aldrich) and 5% fetal bovine serum (FBS; Wisent Bioproducts), which was previously shown to promote high phagocytic activity and MerTK expression.^25^ When indicated, hMGL were polarized by treating them with 20 ng/ml interferon gamma (IFNγ; Peprotech) and 100 ng/ml Pam_3_CSK_4_ (Invivogen) for 48 h or left unpolarized. Fetal microglia (fMGL) were cultured in DMEM (Sigma-Aldrich) supplemented with 1% GlutaMAX^TM^, 1% P/S and 5% FBS. Cells were maintained at 37°C under a 5% CO_2_ atmosphere.

### Generation of iMGL

A number of iPSC lines were used in our study to ensure reproducibility of our findings across lines obtained from different sources: DYR0100 (American Type Cell Collection), KYOU-DXR0109B (American Type Cell Collection), GM25256 (Coriell Institute) and 3450 (generated in-house as previously described^35^). All iPSCs were from individuals with no known neurological diseases. Donor information and reprogramming methods are indicated in Supplementary Table 2. Differentiation of iPSCs into iMGL was carried out following a previously established protocol.^36^ Briefly, haematopoietic progenitor cells were generated from iPSCs using a STEMdiff Hematopoietic kit (STEMCELL Technologies) and cultured in microglia growth medium supplemented with 100 ng/ml interleukin-34, 50 ng/ml tumour growth factor-beta 1 and 25 ng/ml macrophage colony-stimulating factor (Peprotech) for 25 days, following which 100 ng/ml cluster of differentiation 200 (Abcam) and C-X3-C motif chemokine ligand 1 (Peprotech) were also added to the culture. Cells were maintained at 37°C under a 5% CO_2_ atmosphere throughout the protocol. The final culture had a purity of 99% as assessed by PU.1 immunostaining (Supplementary Fig. 1A and B). IMGL expressed a variety of microglia markers at higher levels compared to peripheral blood mononuclear cell-derived macrophages (Supplementary Fig. 1C) or iPSCs.^25^

### Peripheral blood mononuclear cell-derived macrophages

Peripheral blood mononuclear cell-derived macrophages (PBMCs) were isolated from whole blood of healthy individuals (20-year-old female, 24-year-old male, 29-year-old female and 32-year-old male) by Ficoll gradient centrifugation. Monocytes were then collected through magnetic activated bead sorting of CD11b + cells and cultured at a density of 5–10 × 10^5^ cells/cm^2^ (Day 0) in RPMI-1640 (Thermo Fisher Scientific) with 10% FBS, 1% P/S, 1% GlutaMAX^TM^ and 30 ng/ml M-CSF (Peprotech). Cells were matured for 8 days, with media supplementation on Day 4 and Day 7.

### RNA-sequencing

RNA-sequencing (RNAseq) data were obtained from an earlier study.^25^ Quality control of the RNA samples, library preparation and RNAseq were performed by Genome Quebec, Montreal, Canada. The library was generated using a NEBNext^®^ Single Cell/Low Input RNA Library Prep kit (New England Biolabs). RNAseq was performed using an Illumina NovaSeq 6000. The Canadian Center for Computational Genomics’ pipeline GenPipes^37^ was used to align the raw files and quantify the read counts.

### Western blotting

Cells were lysed on ice in a lysis buffer composed of 150 mM NaCl, 50 mM Tris-HCl pH 7.4, 1% Nonidet P-40, 0.1% sodium dodecyl sulfate (SDS) and 5 mM EDTA with protease and phosphatase inhibitors (Thermo Fisher Scientific). Cell lysates were centrifuged at 500*g* for 30 min at 4°C to remove cellular debris. Proteins (25 μg/lane) were separated on SDS-polyacrylamide gels and transferred to polyvinylidene difluoride membranes (Bio-Rad Laboratories). For the detection of α-syn, membranes were fixed in 4% paraformaldehyde solution as previously described.^38^ Membranes were immunoblotted for AXL (AF154 R&D Systems at 1:500), MerTK (ab52968, Abcam at 1:500), phosphorylated MerTK (p186-749, Phosphosolutions at 1:1000), α-syn (ab138501, Abcam 1:5000), S129-phosphorylated α-syn (ab51253, Abcam 1:1000), CSF1R (MAB3291, R&D systems; 1:250) and glyceraldehyde 3-phosphate dehydrogenase (GAPDH; G8795, Sigma Aldrich at 1:5000) overnight at 4°C, and then with horse radish peroxidase-linked secondary antibodies (1:10 000; Jackson Laboratory) for 1 h. Bands were detected by enhanced chemiluminescence with Clarity Max ECL substrates (Bio-Rad Laboratories) using a ChemiDoc Imaging System (Bio-Rad Laboratories). Image analysis was performed using ImageLab 6.0.1 software (Bio-Rad Laboratories).

### Preparation of α-syn preformed fibrils

PFFs of α-syn were generated as previously described^39,40^ with slight modifications. Briefly, glutathione-S-transferase (GST)-tagged human recombinant α-syn were expressed and isolated from *Escherichia coli*. The GST tag was removed and purified α-syn monomers were subjected to endotoxin removal using Pierce^TM^ High-Capacity Endotoxin Removal Resin (Thermo Fisher Scientific) to achieve endotoxin levels <0.3 EU/mg. α-Syn monomers were aggregated on a shaker for 5 days with constant shaking at 1000 rpm and 37°C. Resulting PFFs were subjected to 40–80 cycles of sonication (30 s on/30 s off) using a Zetasizer Nano (Malvern Panalytical) to achieve ∼50–100 nm sizes. Sizes and morphology of PFFs were monitored by electron microscopy and dynamic light scattering (Supplementary Fig. 2A and B). A thioflavin T assay was used to confirm the presence of beta-sheet structure (Supplementary Fig. 2C). All data were validated using α-syn PFFs from at least two different batches.

### Proximity ligation assay

Cells in suspension were incubated with vehicle or α-syn PFFs (1 μM) on ice for 30 min to allow binding, but not internalization, of α-syn PFFs. Unbound α-syn PFFs were washed away, and cells were fixed in 2% formaldehyde solution on ice for 30 min. A proximity ligation assay (PLA) was performed using a Duolink^®^ FlowPLA kit following the manufacturer’s recommendations. The following primary antibodies were used: rabbit anti-α-syn (1:200; ab138501, Abcam), mouse anti-α-syn (1:200; ab1903, Abcam), mouse anti-MerTK (1:1000; 367602, Biolegend) and mouse anti-AXL (1:1000; MAB154, R&D Systems). Antibodies against α-syn were tested on vehicle versus α-syn PFF-treated cells to ensure that they selectively bound to exogenous but not endogenous α-syn (Supplementary Fig. 3). Data acquisition was done on an Attune^TM^ Nxt Flow Cytometer and analysis was done using FlowJo^TM^ software.

### Uptake assay

**Figure 1:**
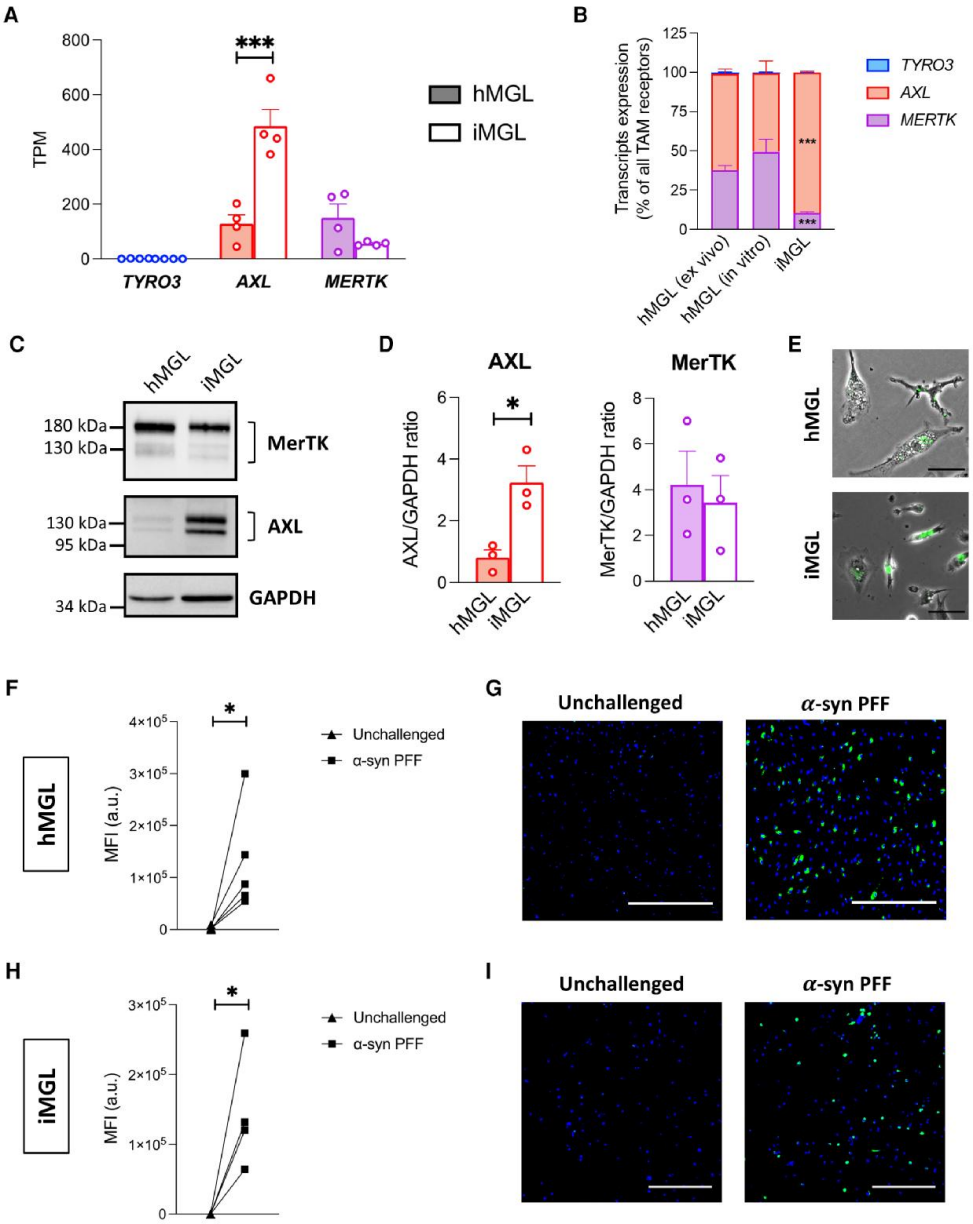
**TAM receptors expression and α-synuclein preformed fibril uptake by human microglia.** All human primary microglia (hMGL) data are from *in vitro* hMGL, unless otherwise specified. (**A** and **B**) RNA-sequencing (RNAseq) assessment of *TYRO3*, *AXL* and *MERTK* expression in hMGL and induced pluripotent stem cell-derived microglia (iMGL). A one-way ANOVA with Sidak’s *post hoc* test was performed for pairwise comparisons between hMGL and iMGL in **A**. TPM = transcripts per million. Mean ± SEM of *n* = 4; ***P < 0.001. A two-way ANOVA with Dunnett’s *post hoc* test was performed to compare *in vitro* hMGL and iMGL to *ex vivo* hMGL in **B**. Mean ± SEM of *n* = 4; ***P < 0.001 versus *ex vivo* hMGL. (**C**) Western blot of MerTK, AXL and GAPDH. (**D**) Quantification of AXL and MerTK expression using GAPDH as a loading control. *t*-tests were performed. Mean ± SEM of *n* = 3; *P < 0.05. (**E**–**I**) hMGL and iMGL were challenged with pHrodo^TM^ Green-labelled α-synuclein (α-syn) preformed fibrils (PFFs) for 2 h. (**E**) Merged phase contrast and green fluorescence images. Scale bar = 50 μm. (**F**) Quantification of mean green fluorescence intensity (MFI) in hMGL culture. A paired *t*-test was performed; *n* = 5; *P < 0.05; a.u. = arbitrary unit. (**G**) Representative fluorescence images of hMGL counterstained with Hoechst 33342 (blue). Scale bar = 500 μm. (**H**) Quantification of MFI in iMGL culture. A paired *t*-test was performed; *n* = 4; *P < 0.05; a.u. = arbitrary unit. (**I**) Representative fluorescence images of iMGL counterstained with Hoechst 33342 (blue). Scale bar = 300 μm.

Human α-syn PFFs, myelin debris^26^ and immunoglobulin G-opsonized red blood cells^41^ (IgG-RBCs) were labelled with pHRodo Green^TM^ STP ester (Thermo Fisher Scientific) and used at the following respective concentrations, which were determined to be non-saturating: 1 μM, 15 μg/ml and 50 000 cells/ml, respectively. Cells were incubated with the labelled substrates for 2 h and then counterstained with Hoechst 33342 (5 μg/ml). Total green fluorescence intensity per cell was quantified using a CellInsight CX5 High Content Screening Platform (Thermo Fisher Scientific). All conditions were assessed in triplicate. Background/autofluorescence subtraction was carried out on all data except those presented in Fig. 1F and H, using fluorescence intensity quantified in unchallenged cells. Internalization of Alexa Fluor 488-conjugated human α-syn PFFs (1 μM) and epidermal growth factor (EGF; 4 μg/ml; Thermo Fisher Scientific) were assessed similarly, except cells were washed with 0.4% trypan blue solution to quench extracellular fluorescence. When indicated, cells were pretreated for 1 h with non-cytotoxic concentrations of UNC2025 (3 μM; Cayman Chemical) or cytochalasin D (1 μM; Sigma Aldrich). At the concentration of 3 μM, UNC2025 inhibited the autophosphorylation of MerTK in iMGL induced by GAS6 + H_2_O_2_ treatment (Supplementary Fig. 4).

### RNA interference

Cells were transfected with siGENOME RISC-Free Control (siCON) or ON-TARGETplus small interfering RNA (siRNA) pools against *MERTK* (siMERTK, at 20 nM) and/or *AXL* (siAXL, at 10 nM) (Horizon Discovery) using Lipofectamine^TM^ RNAiMAX (Thermo Fisher Scientific). The manufacturers’ protocols were followed. When necessary, siCON was used to equilibrate the total amount of siRNA received by cells across experimental conditions.

### Flow cytometry

Cells were blocked with Human TrueStain FcX and TrueStain Monocyte Blocker (Biolegend) and stained with the following antibodies: anti-AXL clone #108724 and anti-MERTK clone #125518 (R&D Systems). Appropriate forward and side scatter profiles were used to exclude debris and doublets from the analysis. Dead cells were excluded based on LIVE/DEAD^TM^ Fixable Aqua (Thermo Fisher Scientific) staining. Readings were done on an Attune^TM^ Nxt Flow Cytometer and analysed/visualized using FlowJo^TM^ software.

### Cell viability

Cell viability was assessed by propidium iodide (PI; Thermo Fisher Scientific) staining unless otherwise specified. Cells were stained in phosphate buffered saline containing 1 μg/ml PI and 5 μg/ml Hoechst 33342 (Thermo Fisher Scientific) and the average number of PI- live cells per condition was determined using a CellInsight CX5 High Content Screening Platform. All conditions were assessed in triplicate. Viability was calculated as follows:


(1) $$Viability_{control}\;(\text{\%})=100\times \;\frac{\#viable\;cells_{control}}{\#viable\;cells_{control}}$$

(2) $$Viability_{experimental}(\text{\%})=100\times \;\frac{\#viable\;cells_{experimental}}{\#viable\;cells_{control}}$$

where


(3) $$\#viable\;cells_{control}=total\;nuclei\;count_{control}-PI\;count_{control}$$

(4) $$\begin{array}{rl} \#viable\;cells_{experimental}= & total\;nuclei\;count_{experimental}\\ & -PI\;count_{experimental} \end{array}$$

### Quantitative reverse transcription PCR

A RNeasy mini kit (Qiagen) was used to extract RNA. Reverse transcription was performed using Moloney murine leukemia virus reverse transcriptase (Thermo Fisher Scientific) and real-time PCR was performed using TaqMan assays (Thermo Fisher Scientific) on a QuantStudio^TM^ 5 real-time PCR system (Thermo Fisher Scientific). The 2^−ΔCt^ method was used to analyse the data using *GAPDH* and tyrosine 3-monooxygenase/tryptophan 5-monooxygenase activation protein zeta (*YWHAZ*) as controls.

### Measurement of cytokine secretion

Concentrations of interleukin-1beta (IL-1β), interleukin-6 (IL-6), interleukin-10 (IL-10) and tumor necrosis factor (TNF) in cell supernatants were measured using the Human Inflammatory Cytokine Cytometric Bead Array Kit (BD Biosciences). Readings were done on an Attune^TM^ Nxt Flow Cytometer.

### Burden analysis of rare variants

Rare variant analysis was performed in the UK Biobank (UKBB) cohort, composed of 602 Parkinson’s disease patients and 15 000 randomly sampled controls, and the Accelerating Medicines Partnership-Parkinson Disease (AMP-PD) initiative cohort, composed of 2341 Parkinson’s disease patients and 3486 controls. All Parkinson’s disease patients were diagnosed according to either the UK Brain Bank criteria^42^ or the Movement Disorders Society criteria.^43^ Only individuals with European ancestry were included. For the UKBB cohort, whole-exome sequencing data were used. Quality control was performed both at the individual and variant levels using Genome Analysis Toolkit (GATK, v3.8) and plink, with a minimum depth of coverage = 10 and GQ = 20 as described previously.^44^ For the AMP-PD cohort, all available whole-genome sequencing data were used for which the quality control processes at the individual and variant levels have previously been described (https://amp-pd.org/whole-genome-data). Associations between variants and Parkinson’s disease were tested using the optimized sequence Kernel association test (SKAT-O).^45^ Variants were categorized into (i) rare (minor allele frequency <1%) non-synonymous variants; (ii) functional variants (nonsynonymous, stop/frameshift and splicing); and (iii) variants with a combined annotation dependent depletion (CADD) score of ≥20, representing the top 1% of potentially deleterious variants. A meta-analysis of the two cohorts was run using the metaSKAT package.^46^

### Analysis of single-nuclei RNA-sequencing data

Single-nuclei RNAseq data for human substantia nigra were obtained from Kamath *et al*.^47^ and accessed through GEO:178265. The dataset included eight non-neurological control donors, seven Parkinson’s disease patients and three Lewy body dementia patients. All patients were pathologically confirmed to present moderate to prominent loss of pigmentation in the substantia nigra.^47^ The microglia population was subtracted from the main object using metadata information provided by the authors at the Broad Institute Single Cell Portal https://singlecell.broadinstitute.org/single_cell/study/SCP1768/. The subtracted microglia population was subjected to the standard Seurat pipeline for dimensionality reduction, gene expression normalization, clustering and differential expression analysis. Twenty clusters expressing microglia canonical markers were identified (Supplementary Fig. 5), among which Clusters 8, 12, 16 and 17 showed enrichment in stress response genes typically associated with tissue dissociation procedures (Supplementary Table 3). Those clusters, along with Cluster 11 with macrophage marker expression (Supplementary Table 3), were removed from further analysis. Differentially expressed genes (DEGs) between Parkinson’s disease/Lewy body dementia and control microglia were identified using |log_2_(fold change)| > 0.25 and adjusted *P*-value < 0.05 as cut-offs. Microglia from control, Parkinson’s disease and Lewy body dementia samples were integrated using the canonical correlation analysis (CCA) of the Seurat pipeline. Microglia clusters identified post-integration were used to generate Supplementary Figs 11 and 12.

### Preparation of human brain tissue lysates

Human substantia nigra tissues were from the Netherlands Brain Bank (NBB), Netherlands Institute for Neuroscience, Amsterdam, Netherlands (open access: www.brainbank.nl), and derived from nine Lewy body dementia patients and nine control donors. All materials were collected from donors for or from whom a written informed consent for a brain autopsy and the use of the material and clinical information for research purposes had been obtained by the NBB. Since availability of substantia nigra from non-neurological controls was limited, substantia nigra from control donors presenting non-synucleinopathy neurological disorders sparing the substantia nigra were used. All control donors had normal appearing, pigmented substantia nigra. Clinicopathological information about the donors is presented in Supplementary Table 4. Detailed clinicopathological information is available upon request. Human cortex tissues were from the Mayo Clinic Brain Bank, Jacksonville, USA, and derived from five Lewy body dementia patients and five control donors devoid of Lewy body disease, from or for whom a written informed consent for a brain autopsy and the use of the material and clinical information for research purposes had been obtained. Clinicopathological information about the donors is presented in Supplementary Table 5.

Tissues were dounce-homogenized in Tris-buffered saline with protease and phosphatase inhibitors. Samples were further diluted in Tris-buffered saline with 5% SDS, 8 M urea, protease and phosphatase inhibitors for protein extraction. Insoluble materials were removed by centrifugation at 13 000*g* for 10 min.

### Statistical analyses

Statistical analyses were performed using GraphPad Prism 9.0 software. A *t*-test was used to compare the mean of two groups of data. A one-way ANOVA was used to compare the mean of three or more groups of data. When the assumptions of a *t*-test (normal distribution) or a one-way ANOVA (normal distribution and homoscedasticity) were not met, a Mann–Whitney or Kruskal–Wallis test was used instead, respectively. Paired or repeated measure analyses were performed for experiments involving hMGL from the same individuals or iMGL from the same differentiation batches subjected to two or more treatment conditions. *P*-values were adjusted using appropriate *post hoc* tests in the case of multiple comparisons. Mean and standard error of the mean (SEM) of biological replicates (*n*) are plotted in all graphs unless otherwise indicated. A *P* < 0.05 was considered statistically significant. HMGL obtained from independent donors and iMGL generated at different points in time were considered biological replicates.

## Results

### HMGL and iMGL express MerTK and internalize α-syn preformed fibrils

We first aimed to assess the MerTK-dependence of α-syn PFF uptake by human microglia. Given the limited access to hMGL, the potential of iMGL as an alternative model to investigate the role of MerTK in α-syn PFF uptake was evaluated. RNAseq and western blotting of TAM receptor expression revealed that both AXL and MerTK are expressed in hMGL and iMGL (Fig. 1A–D). Interestingly, AXL expression was significantly higher in iMGL compared to hMGL, whereas MerTK expression was similar between both cell types (Fig. 1A–D). As expected, *TYRO3* expression was low in both models (Fig. 1A and B). Microglia undergo a major transcriptomic change from their freshly isolated state (*ex vivo*) to a cultured state (*in vitro*).^23,25^ RNAseq assessment of TAM receptor expression revealed a similar *MERTK*:*AXL* expression ratio between *ex vivo* and *in vitro* hMGL, whereas this ratio was lower in iMGL (Fig. 1B). This suggests the relative expression profile of TAM receptors in *in vitro* hMGL is representative of their native profile, whereas the TAM receptor expression profile in iMGL is distinct. Given these results, subsequent experiments were carried out in iMGL, with validation of key findings in hMGL.

pHrodo^TM^ dyes are fluorogenic dyes that fluoresce when present in an acidic milieu such as phagosomes and lysosomes; and they have previously been demonstrated to be useful tools for quantifying the rate of phagocytosis.^26,48^ Following incubation of iMGL with pHrodo^TM^ Green-labelled α-syn PFFs, a dose-dependent and time-dependent increase in green fluorescence intensity per cell was quantified (Supplementary Fig. 6A and B). A concentration of 1 μM was non-saturating at the 2-h time point (Supplementary Fig. 6A and B) and resulted in readily quantifiable fluorescence intensity in both hMGL (Fig. 1E–G) and iMGL cultures (Fig. 1E, H and I).

### α-Syn preformed fibrils bound on microglia cell surface are in close proximity to MerTK

**Figure 2:**
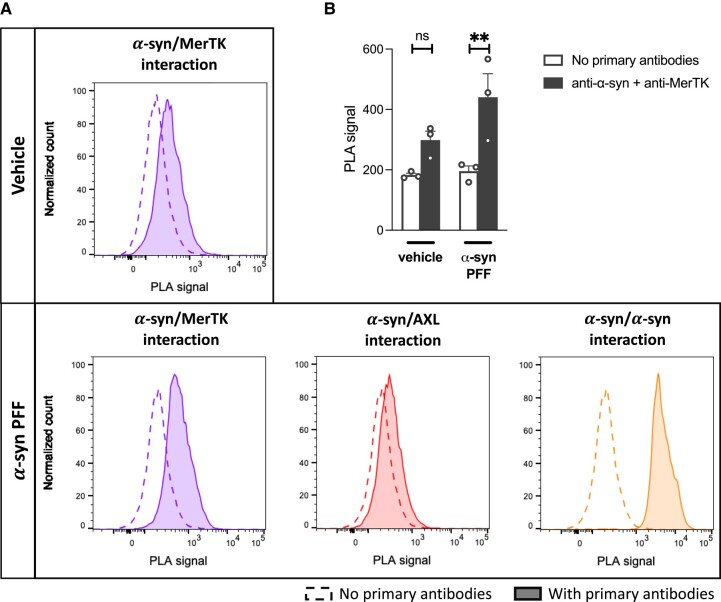
**Interaction between α-synuclein preformed fibrils and MerTK.** Induced pluripotent stem cell-derived microglia (iMGL) were incubated with α-synuclein (α-syn) preformed fibrils (PFFs) or vehicle on ice for 30 min and a proximity ligation assay (PLA) was performed. (**A**) Representative flow cytometry plots. (**B**) Quantification of the PLA signals. A repeated measure one-way ANOVA was performed followed by Sidak’s *post hoc* test. Mean ± SEM of *n* = 3; **P < 0.01.

PLA is a highly sensitive and specific method of assessing interactions between two proteins. A pair of antibodies raised in two different species are used to detect close proximity (<40 nm) of their respective target proteins in cellular systems.^49^ IMGL were incubated on ice with α-syn PFFs, and flow cytometry-based PLA was performed to assess the interactions between α-syn PFFs and MerTK on cell surfaces. As a positive control, interactions between α-syn proteins were shown to emit high PLA signals when two different antibodies against α-syn were used, confirming α-syn/α-syn interactions within fibrils (Fig. 2A). A positive PLA signal was also detected when interaction between α-syn and MerTK was assessed (Fig. 2A and B). In contrast, signal was minimal when interaction between α-syn and AXL was measured (Fig. 2A). Overall, these data demonstrate that α-syn PFFs bound to the cell surface are in close proximity to MerTK, suggestive of an interaction between α-syn PFFs and MerTK prior to their internalization.

### Pharmacological inhibition of MerTK results in decreased α-syn preformed fibrils uptake by microglia

**Figure 3:**
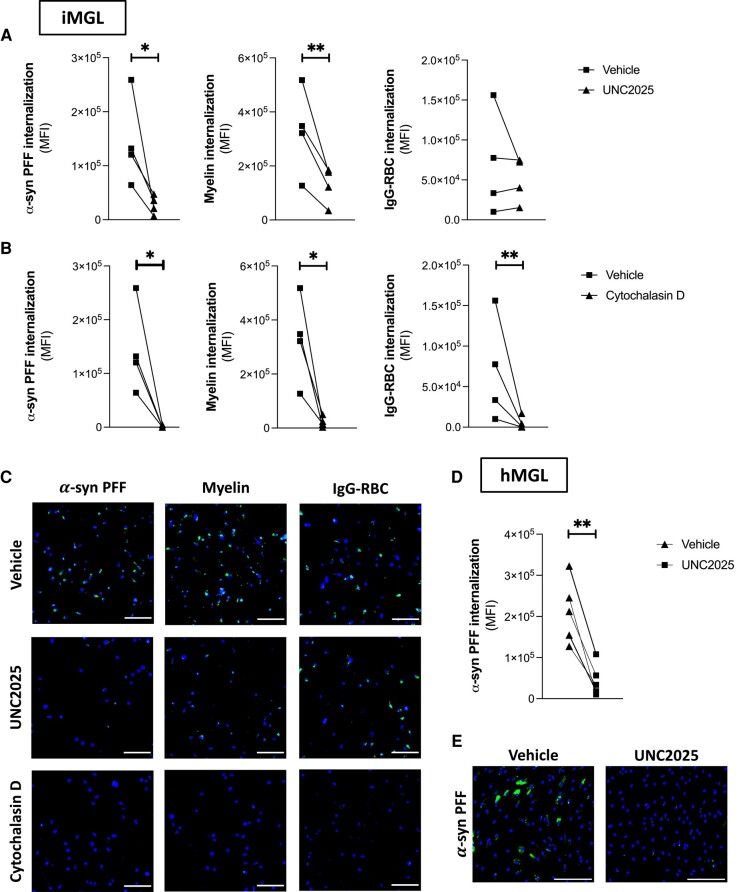
**Effect of UNC2025 on microglial uptake of α-synuclein preformed fibrils.** Microglia were pretreated with vehicle, UNC2025 (3 μm) or cytochalasin D (1 μm) for 1 h, then challenged with pHrodo^TM^ Green-labelled α-synuclein (α-syn) preformed fibrils (PFFs), myelin debris or IgG-opsonized red blood cells (IgG-RBC) for 2 h. (**A** and **B**) Quantification of mean green fluorescence intensity (MFI) in induced pluripotent stem cell-derived microglia (iMGL) culture. Paired *t*-tests were performed; *n* = 4; *P < 0.05, **P < 0.01. (**C**) Representative images of iMGL counterstained with Hoechst 33342 (blue). Scale bar = 150 μm. (**D**) Quantification of mean green fluorescence intensity (MFI) in human primary microglia (hMGL) culture. A paired *t*-test was performed; *n* = 5; **P < 0.01. (**E**) Representative images of hMGL counterstained with Hoechst 33342 (blue). Scale bar = 200 μm.

The selective MerTK inhibitor UNC2025^50^ was used to investigate the involvement of MerTK in α-syn fibril internalization by microglia. A 1-h pretreatment with UNC2025 reduced iMGL uptake of pHrodo^TM^ Green-labelled α-syn PFFs by ∼79% (Fig. 3A–C). UNC2025 also decreased pHrodo^TM^ Green-labelled myelin uptake as previously described,^26^ but had no effect on pHrodo^TM^ Green-labelled IgG-RBC uptake, a process mediated by Fc gamma receptors and thus independent of MerTK^27,51^ (Fig. 3A–C). In contrast, the actin polymerization inhibitor cytochalasin D non-selectively inhibited the internalization of all three substrates (Fig. 3A–C). UNC2025 also decreased hMGL uptake of pHrodo^TM^ Green-labelled α-syn PFFs by ∼79% (Fig. 3D and E), providing validation that MerTK kinase activity is essential for the internalization of α-syn fibrils by hMGL.

To ensure that results were not influenced by the use of a pH-sensitive dye, additional tests were performed with Alexa Fluor 488-labelled α-syn PFFs. Similar to findings with pHrodo^TM^ Green-labelled α-syn PFFs, UNC2025 also decreased the internalization of Alexa Fluor 488-labelled α-syn PFFs by iMGL (Supplementary Fig. 7A and B), confirming that MerTK appears to mediate the internalization of α-syn PFFs regardless of the conjugated dyes. UNC2025 did not affect the internalization of Alexa Fluor 488-labelled EGF, which is known to be mediated by epidermal growth factor receptors^52^ (EGFRs; Supplementary Fig. 7A and B). All subsequent uptake assays were performed using pHrodo^TM^ Green-labelled α-syn PFFs.

### *MERTK* knockdown results in decreased α-syn preformed fibril uptake by microglia

**Figure 4:**
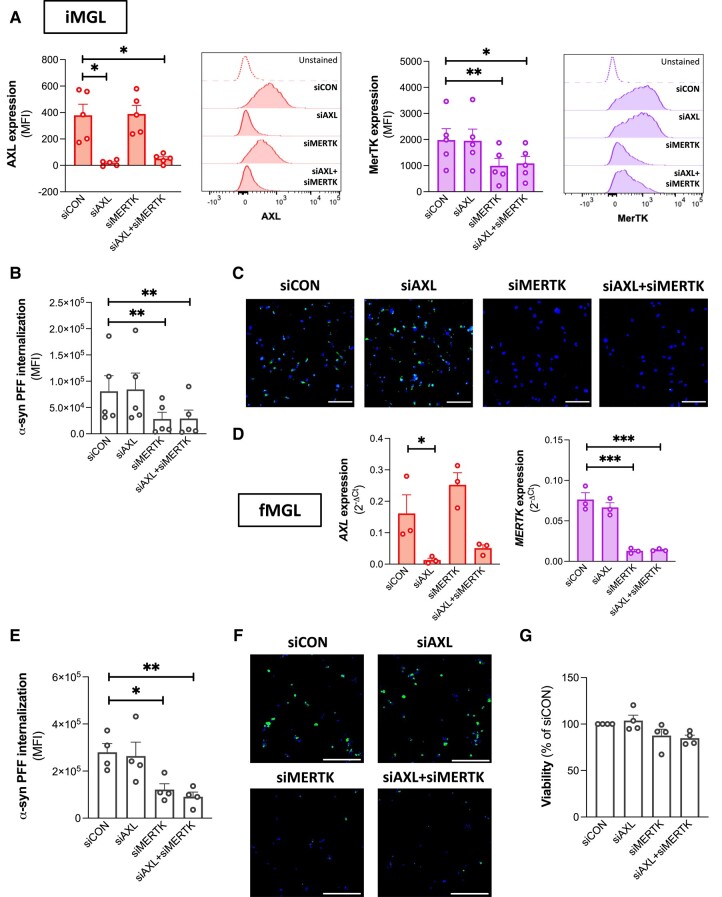
**Effect of *AXL*/*MERTK* knockdown on microglial uptake of α-synuclein preformed fibrils.** Microglia were transfected with siCON, siAXL and/or siMERTK. (**A**) Cell surface expression of AXL and MerTK assessed by flow cytometry on live induced pluripotent stem cell-derived microglia (iMGL) 48 h following transfection. Repeated measure one-way ANOVA were performed followed by Dunnett’s *post hoc* test. Mean ± SEM of *n* = 5; *P < 0.05, **P < 0.01; MFI = median fluorescence intensity. (**B**) Quantification of mean green fluorescence intensity (MFI) in induced pluripotent stem cell-derived microglia (iMGL) culture challenged with pHrodo^TM^ Green-labelled α-synuclein (α-syn) preformed fibrils (PFFs) for 2 h. A repeated measure one-way ANOVA was performed followed by Dunnett’s *post hoc* test. Mean ± SEM of *n* = 5; **P < 0.01. (**C**) Representative images of iMGL challenged with pHrodo^TM^ Green-labelled α-syn PFFs for 2 h and counterstained with Hoechst 33342 (blue). Scale bar = 150 μm. (**D**) qRT-PCR assessment of *AXL* and *MERTK* expression in fetal microglia (fMGL) 48 h following transfection. Repeated measure one-way ANOVA were performed followed by Dunnett’s *post hoc* test. Mean ± SEM of *n* = 3; *P < 0.05, ***P < 0.001. (**E**) Quantification of mean green fluorescence intensity (MFI) in fMGL culture challenged with pHrodo^TM^ Green-labelled α-syn PFFs for 2 h. A repeated measure one-way ANOVA was performed followed by Dunnett’s *post hoc* test. Mean ± SEM of *n* = 4; *P < 0.05, **P < 0.01. (**F**) Representative fluorescence images of fMGL challenged with pHrodo^TM^ Green-labelled α-syn PFFs for 2 h and counterstained with Hoechst 33342 (blue). Scale bar = 200 μm. (**G**) Viability of fMGL 48 h following transfection. A repeated measure one-way ANOVA was performed followed by Dunnett’s *post hoc* test. Mean ± SEM of *n* = 4.

Small-interfering RNA-mediated knockdown experiments were next carried out to confirm that our earlier findings were a consequence of MerTK inhibition and not an off-target effect of UNC2025 on other tyrosine kinases. IMGL were transfected with siRNAs against *AXL* and/or *MERTK* and replated for the assessment of α-syn PFF uptake. siAXL and siMERTK significantly decreased the respective surface expression of AXL and MerTK on live iMGL after 48 h (Fig. 4A). Knockdown of *AXL* had no marked impact on α-syn PFF internalization by iMGL, unlike *MERTK* knockdown, which resulted in a ∼71% decrease in internalization (Fig. 4B and C). Knockdown of both *AXL* and *MERTK* together did not further affect α-syn PFF uptake compared to *MERTK* knockdown alone (Fig. 4B and C). As postnatal hMGL are difficult to transfect, cells from fetal sources were used to validate the effect of *MERTK* knockdown on α-syn fibril internalization by human microglia. fMGL expressed MerTK and AXL at similar levels to hMGL (Supplementary Fig. 8A–C), and UNC2025 decreased their uptake of α-syn PFFs by ∼64% (Supplementary Fig. 8D and E). *AXL* knockdown did not affect α-syn PFF uptake by fMGL, whereas *MERTK* knockdown did (by ∼56%; Fig. 4D–F). Neither *AXL* nor *MERTK* knockdown affected fMGL viability (Fig. 4G), ruling out the possibility that a viability effect was downregulating PFF internalization. Altogether, these findings implied that MerTK but not AXL is essential for α-syn fibril uptake by human microglia.

Previously, it has been shown that combined stimulation of microglia with IFNγ and a TLR agonist, the so-called ‘M1’ polarization, results in a pro-inflammatory phenotype characterized by decreased expression of MerTK and lower internalization of myelin debris.^26^ Polarization of hMGL with IFNγ and the TLR2/1 agonist Pam_3_CSK_4_ was confirmed to increase the expression of inflammatory markers such as human leukocyte antigen-DR (*HLA-DR*), *TNF* and C-X-C motif chemokine ligand 10 (*CXCL10*), and to decrease *IL10* expression compared to the unpolarized state (Supplementary Fig. 9A). Expression of genes encoding MerTK and its activating ligands GAS6 and PROS1 were decreased in cells polarized with IFNγ and Pam_3_CSK_4_ whereas AXL expression was unchanged (Supplementary Fig. 9A). This decreased expression of *MERTK*, *GAS6* and *PROS1* was associated with significantly lower uptake of α-syn PFFs (Supplementary Fig. 9B and C), further supporting the notion that MerTK alone, not AXL, mediates α-syn fibril uptake by human microglia.

### MerTK-mediated α-syn preformed fibril uptake has an immunomodulatory effect on microglia

**Figure 5:**
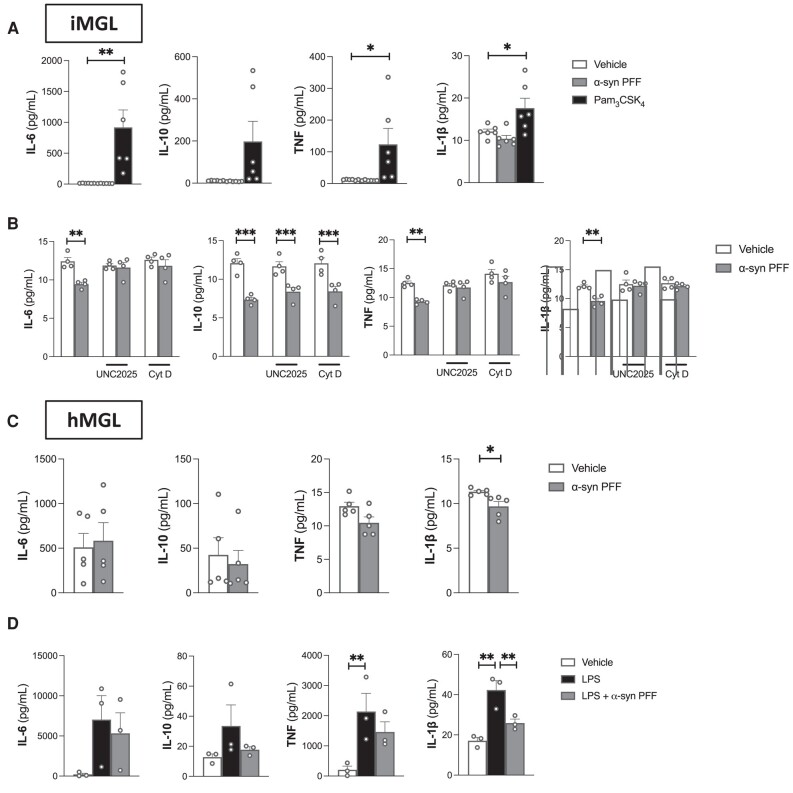
**Inflammatory response of human microglia to α-synuclein preformed fibrils.** Microglia were treated for 24 h with vehicle, Pam_3_CSK_4_ (100 ng/ml), lipopolysaccharide (LPS; 100 ng/ml) and/or α-synuclein (α-syn) preformed fibrils (PFFs) (1 μm). (**A**) Cytokine secretion from induced pluripotent stem cell-derived microglia (iMGL). Repeated measure one-way ANOVA were performed followed by Dunnett’s *post hoc* test. Mean ± SEM of *n* = 6; *P < 0.05, **P < 0.01. (**B**) Cytokine secretion from iMGL pretreated or not with UNC2025 (3 μm) or cytochalasin D (cyt D; 1 μm) for 1 h prior to vehicle/α-syn PFF treatment. Repeated measure one-way ANOVA were performed followed by Sidak’s *post hoc* test. Mean ± SEM of *n* = 4; **P < 0.01, ***P < 0.001. (**C**) Cytokine secretion from hMGL. Paired *t*-tests were performed. Mean ± SEM of *n* = 6; *P < 0.05. (**D**) Cytokine secretion from human primary microglia (hMGL). Repeated measure one-way ANOVA were performed followed by Sidak’s *post hoc* test. Mean ± SEM of *n* = 3; **P < 0.01. IL-1β = interleukin-1β; IL-6 = interleukin-6; IL-10 = interleukin-10; TNF = tumor necrosis factor.

Phagocytic processes involving MerTK are known to be non-inflammatory and can even induce immunosuppression.^53^ With pharmacological and knockdown assays pointing towards MerTK being a central mediator of α-syn fibril uptake, the effects of α-syn PFFs on immune pathways was investigated. Pam_3_CSK_4_ significantly increased the secretion of IL-6 and TNF by iMGL, confirming that these cells can be activated to secrete cytokines (Fig. 5A). Consistent with the idea that MerTK-mediated phagocytosis is immunologically silent, a 24-h treatment with α-syn PFFs did not result in increased iMGL secretion of IL-6, TNF, IL-10 or IL-1β (Fig. 5A). Interestingly, a modest but statistically significant decrease in the secretion of those cytokines was observed following α-syn PFF treatment (Fig. 5B), indicative of an immunomodulatory effect. The α-syn PFF-induced decrease in IL-6, TNF and IL-1β was prevented by a 1-h pretreatment of iMGL with UNC2025 or cytochalasin D (Fig. 5B), suggesting that this immunomodulatory effect is dependent on MerTK-mediated α-syn PFF internalization. The decrease in IL-10 was not prevented by UNC2025 or cytochalasin D pretreatment (Fig. 5B), suggesting that this effect of α-syn PFFs is independent from their internalization. Treatment of hMGL with α-syn PFFs also did not elicit an increase in IL-6, TNF, IL-10 or IL-1β secretion (Fig. 5C). IL-1β secretion by hMGL was significantly inhibited by α-syn PFFs (Fig. 5C). In hMGL, α-syn PFFs were also found to inhibit lipopolysaccharide (LPS)-induced IL-1β secretion (Fig. 5D).

Expression of a wider range of cytokines was assessed in iMGL by qRT-PCR following 3 h, 1 day and 7 days of α-syn exposure. In contrast to Pam_3_CSK_4_, neither the monomeric nor fibrillar forms of α-syn increased the transcriptional expression of cytokines (Supplementary Fig. 10). To summarize, α-syn did not induce an inflammatory response of human microglia similar to that triggered by TLR stimulation.

### Rare deleterious *MERTK* variants are associated with Parkinson’s disease

To further examine the potential role of MerTK-mediated phagocytosis in synucleinopathies, genetic associations between rare *MERTK* variants and Parkinson’s disease were investigated by burden analysis in two independent patient cohorts: UKBB and AMP-PD. A significant association between *MERTK* variants with CADD score ≥20 (representing the top 1% of potentially deleterious variants) and Parkinson’s disease was found in the UKBB cohort (*P* = 0.002; Table 1). However, this association was not observed in the AMP-PD cohort or following a meta-analysis of both cohorts (Table 1). This could be a result of specific variants associated with Parkinson’s disease being underrepresented in the AMP-PD cohort. No significant associations were found between *AXL* variants and Parkinson’s disease (Table 1). Overall, these results suggest that some rare, functionally deleterious *MERTK* variants may be associated with Parkinson’s disease, however these findings require additional replications.

**Table 1:** Burden analysis of rare variants in Parkinson’s disease

Gene	All non-synonymous, *P*			All functional, *P*			CADD score ≥20, *P*		
	UKBB	AMP-PD	Meta-analysis	UKBB	AMP-PD	Meta-analysis	UKBB	AMP-PD	Meta-analysis
*AXL*	0.84	0.27	0.48	0.83	0.27	0.48	0.45	0.85	0.54
*MERTK*	0.2	0.92	0.81	0.21	1	0.78	**0**.**002**	0.67	0.28

Bold text represents statistically significant value (*P* < 0.05). AMP-PD = Accelerating Medicines Partnership-Parkinson’s Disease; CADD = combined annotation dependent depletion; UKBB = UK Biobank.

### MerTK protein expression is unaltered in brain lysates of Lewy body dementia patients

**Figure 6:**
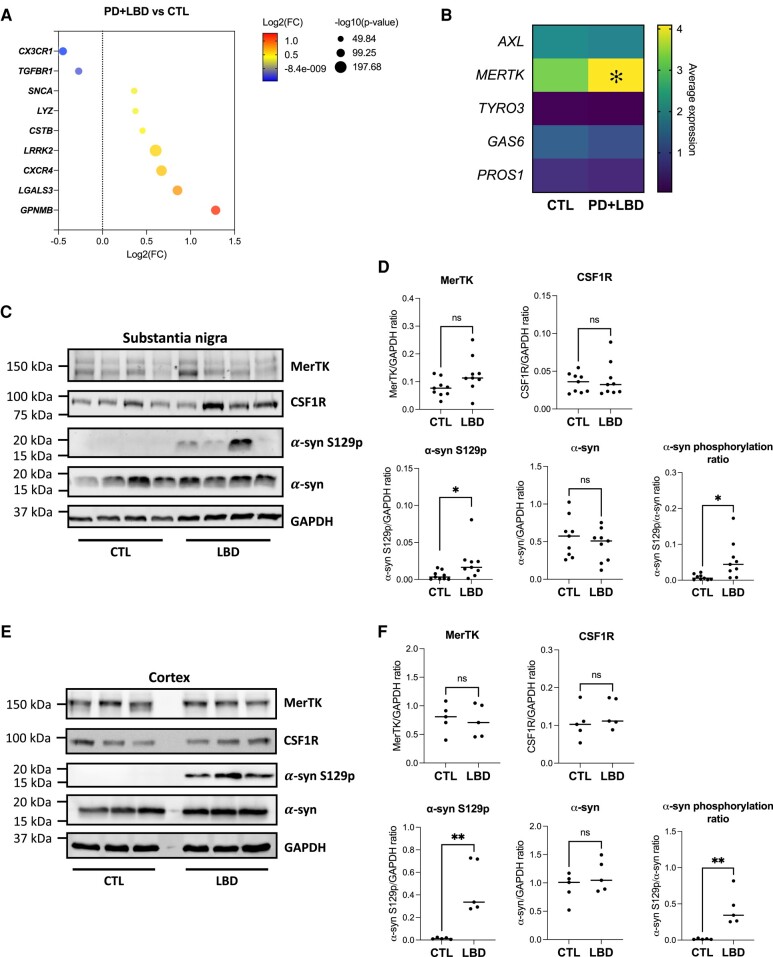
**MerTK expression in Parkinson’s disease and Lewy body dementia.** (**A** and **B**) Single-nuclei RNA-sequencing data from Kamath *et al.*^47^ were used to compare microglia from substantia nigra of seven Parkinson’s disease (PD) patients and three Lewy body dementia (LBD) patients versus eight control (CTL) donors. (**A**) Dot plot depicting examples of identified differentially expressed genes (DEGs) between PD + LBD and CTL. (**B**) Heatmap showing the average expression of genes encoding TAM receptors and their activating ligands. *P < 0.05 in DEG analysis. (**C** and **D**) Western blotting using human substantia nigra tissues of LBD and CTL donors. (**C**) Representative blots. Proteins were extracted in the presence of SDS and urea to solubilize α-synuclein (α-syn) aggregates. (**D**) Quantification of MerTK, CSF1R, phosphorylated α-syn (S129p) and α-syn expression. Mann–Whitney tests were performed; *n* = 9; ns = non-significant; *P < 0.05. (**E** and **F**) Western blotting using human cortex of LBD and CTL donors. (**E**) Representative blots. Proteins were extracted in the presence of SDS and urea to solubilize α-syn aggregates. (**F**) Quantification of MerTK, CSF1R, phosphorylated α-syn (S129p) and α-syn expression. Mann–Whitney tests were performed; *n* = 5; ns = non-significant; **P < 0.01.

Following the observation that rare genetic variants of *MERTK* are potentially associated with Parkinson’s disease, the possibility that the MerTK pathway is dysregulated in the Parkinson’s disease substantia nigra was investigated using a previously published post-mortem snRNAseq dataset.^47^ DEG analysis of microglia from Parkinson’s disease (*n* = 7) or Lewy body dementia (*n* = 3) substantia nigra tissues compared to those of non-neurological controls (*n* = 8) revealed a decrease in expression of the homeostatic markers C-X3-C motif chemokine receptor 1 (*CX3CR1*) and transforming growth factor beta receptor 1 (*TGFBR1*), accompanied by an increase in the expression of specific neurodegeneration-associated genes that included C-X-C motif chemokine receptor 4 (*CXCR4*) and glycoprotein NMB (*GPNMB*), in the diseased group (Fig. 6A). Expression of *SNCA* and leucine-rich repeat kinase 2 (*LRRK2*) with known genetic association to Parkinson’s disease was also significantly elevated in microglia from Parkinson’s disease/Lewy body dementia patients (Fig. 6A). Consistent with our *in vitro* data, genes encoding inflammatory cytokines did not figure among the DEGs (Supplementary Table 6). Most importantly, evaluation of genes encoding TAM receptors and their activating ligands revealed *MERTK* expression to be modestly higher in microglia from diseased patients compared to controls (fold change = 1.2, *P* = 5.08 × 10^−21^; Fig. 6B and Supplementary Table 6). *MERTK* was ubiquitously expressed across all microglia clusters identified in the dataset (Supplementary Fig. 11). Some clusters predominantly composed of microglia from Parkinson’s disease/Lewy body dementia patients had particularly high expression of *MERTK* (Clusters 7, 8, 10, 15 and 16; Supplementary Figs 11 and 12). Aside from microglia which had the highest expression of *MERTK*, macrophages, astrocytes and endothelial cells expressed *MERTK* in the substantia nigra (Supplementary Fig. 13). No significant difference in *MERTK* expression was observed between control and Parkinson’s disease/Lewy body dementia patients when DEG analysis was carried out on all cell types combined (Supplementary Table 7).

Analysis of transcriptional changes following *in vitro* exposure of iMGL to α-syn PFFs revealed increased *CXCR4* and *GPNMB* expression after 1 and 7 days, respectively (Supplementary Fig. 10). In contrast, expression of homeostatic markers such as *CX3CR1* and *TGFBR1*, as well as *MERTK* were unaffected by *in vitro* α-syn PFF exposure (Supplementary Fig. 10), suggesting α-syn fibrils are direct drivers of some, but not all, microglial transcriptomic changes in late-stage Parkinson’s disease/Lewy body dementia. Transcriptional changes in patient microglia could be a consequence of other processes such as neuronal dysfunction or degeneration.

Upregulation of MerTK protein expression in synucleinopathies was next verified by western blotting using substantia nigra tissues from nine Lewy body dementia patients and nine control donors. Significantly higher amounts of α-syn phosphorylated at serine 129 (S129p), an indicator of α-syn fibril accumulation, but not total α-syn, were detected in substantia nigra lysates of Lewy body dementia patients compared to controls (Fig. 6C and D and Supplementary Fig. 14). This is consistent with previous observations.^54,55^ The accumulation of α-syn S129p was unaccompanied by upregulation in MerTK protein expression in whole tissue lysates (Fig. 6C and D). Similar observations were made using cortex tissues from five Lewy body dementia patients and five non-neurological control donors (Fig. 6E and F and Supplementary Fig. 15). Altogether, the data suggest a lack of substantial upregulation in MerTK expression in synucleinopathies such as Lewy body dementia.

## Discussion

In summary, our study identified MerTK as an essential receptor that mediates α-syn fibril uptake by human microglia. Using orthogonal assays, including pharmacological inhibition of MerTK kinase activity, *MERTK* downregulation induced by phenotypic polarization and *MERTK* knockdown by siRNA, we show that α-syn fibril internalization by human microglia is dependent on MerTK. MerTK-mediated internalization of α-syn PFFs was not observed to induce the expression and secretion of inflammatory cytokines. Moreover, *MERTK* mRNA expression was found to be only modestly increased in microglia from Parkinson’s disease/Lewy body dementia patients compared to control donors. At the whole tissue level, accumulation of α-syn S129p in the brains of Lewy body dementia patients was unaccompanied by MerTK protein upregulation. *MERTK* mRNA upregulation in microglia might not have translated into higher protein expression, or was too modest to be reflected at the tissue level given that other cell types such as astrocyte express MerTK. Our data exclude the possibility that a dysregulated MerTK pathway is responsible for α-syn fibril accumulation in Parkinson’s disease/Lewy body dementia, since no downregulation of MerTK expression was observed. However, specific genetic variants that are deleterious to MerTK function could be associated with Parkinson’s disease according to the result of our burden analysis. Overall, our findings imply that therapeutic upregulation of this pathway in synucleinopathies might promote α-syn fibril clearance without inducing inflammation.

Phagocytosis of cellular debris is a complex process that involves multiple cell surface proteins, some mediating tethering of the target and some initiating engulfment through cytoskeletal remodelling. Pattern recognition receptors such as TLRs can come into contact with their agonistic motifs during this process and trigger an inflammatory response.^56^ α-Syn has been repeatedly observed to induce inflammatory cytokine release from murine microglia,^16-20^ albeit with some contradictory findings.^57^ With regards to human iMGL, Trudler *et al.*^58^ previously observed inflammasome activation and IL-1β secretion following cell treatment with non-sonicated large size α-syn PFFs (>2000 nm), whereas Rostami *et al.*^14^ failed to observe IL-1β secretion from iMGL following sonicated, smaller α-syn PFFs treatment. With regards to hMGL, non-sonicated α-syn PFFs were previously shown by Pike *et al.*^59^ to induce inflammasome activation and subsequent IL-1β release from hMGL. In our study, α-syn PFFs sonicated to ∼50–100 nm in size were employed because of the demonstrated pathogenicity and propensity for propagation of smaller PFFs.^60,61^ These small PFFs were actively phagocytosed but failed to induce IL-1β secretion from hMGL or iMGL. Notably, microglia in both the Pike *et al.*^59^ and Trudler *et al.*^58^ studies were cultured in the presence of granulocyte-macrophage colony-stimulating factor (GM-CSF), which might have influenced microglia response to α-syn PFFs. Finally, Pike *et al.*^59^ used saturating concentrations of α-syn PFFs (5–20 μM), which might have resulted in stress-induced non-specific activation of microglia.

Although the TAM receptors AXL and MerTK were thought to have redundant roles in phagocytosis, MerTK but not AXL has been demonstrated to mediate homeostatic phagocytosis. Daily clearance of photoreceptor outer segments by retinal pigment epithelial cells in the retina and apoptotic germ cells by Sertoli cells in the testis are mediated by MerTK,^62^ without which accumulation of cellular debris leads to tissue degeneration.^29,63-65^ AXL and MerTK expression are also differentially regulated by tolerogenic and inflammatory stimuli: whereas MerTK expression is increased by treatment with glucocorticoids, AXL is upregulated and MerTK downmodulated following lipopolysaccharide or polyinosinic:polycytidylic treatment of macrophages.^66,67^ Here, we observed that microglial uptake of α-syn fibrils is dependent on MerTK but not AXL despite both receptors being expressed by human microglia. Consistent with the *in vitro* observations, only *MERTK* but not *AXL* variants were genetically associated with Parkinson’s disease cases in the UKBB cohort.

In a study that quantified the presence of α-syn inclusions in different cell types of the olfactory bulb of Parkinson’s disease patients, ∼8% of microglia have been shown to contain inclusions, a strikingly high percentage considering that ∼9% of neurons were found to contain inclusions in the same study.^68^ The main source of microglia-internalized α-syn in the Parkinson’s disease brain remains unclear. α-Syn has been detected in the cerebrospinal fluid and brain interstitial fluid, suggesting the protein is released by neurons into the extracellular space through secretion or cell death.^69,70^ The current study therefore focused on the role of MerTK in internalization of extracellular α-syn fibrils. However, MerTK could well be mediating α-syn internalization through phagocytosis of synapses^71^ and apoptotic neurons,^72^ limiting neuronal spread of α-syn.

MerTK has been receiving increasing attention as a potential therapeutic target in proteinopathies over recent years. In the APP/PS1 mouse model of Alzheimer’s disease, Axl and MerTK have been shown to be important for microglia migration to amyloid plaques.^73^ Genetic ablation of *Axl* and/or *Mertk* led to accelerated death of the animals,^74^ indicating a protective role for these receptors against disease progression. Longitudinal studies revealed that higher circulating levels of cleaved, soluble TAM receptors and their ligand GAS6 in the CSF are associated with better cognitive outcome in Alzheimer’s disease.^75,76^ To our knowledge, the current study represents the first *in vitro* demonstration of MerTK involvement in protein aggregate internalization. Pharmacological targeting of MerTK is difficult because of its wide expression and pleiotropic roles. Enhancement of MerTK expression or function in the brain is likely to increase phagocytosis of all MerTK targets, including myelin, synapses, apoptotic cells and even live neurons in a process termed phagoptosis.^77^ Bispecific antibodies and hybrid proteins with affinities to both MerTK and amyloid beta have been designed to specifically target amyloid beta to MerTK-mediated, non-inflammatory phagocytosis in Alzheimer’s disease.^78,79^ Similar strategies could be adopted to selectively enhance α-syn clearance through MerTK in synucleinopathies.

In conclusion, we report for the first time a role for MerTK in the uptake of α-syn fibrils by human microglia. This pathway could be leveraged to promote α-syn fibril clearance in synucleinopathies such as Parkinson’s disease without inducing an inflammatory response from microglia.

## Supplementary Material

awad298_Supplementary_Data

## Data Availability

RNAseq data from Dorion *et al.*^25^ are available through Gene Expression Omnibus (GSE228755). snRNAseq data from Kamath *et al.*^47^ are available through the Gene Expression Omnibus (GSE178265).

## References

[1] Klein C , Westenberger A. Genetics of Parkinson's disease. Cold Spring Harb Perspect Med. 2012;2:a008888. 22315721 10.1101/cshperspect.a008888 PMC3253033

[2] Braak H , Rüb U, Gai WP, Del Tredici K. Idiopathic Parkinson's disease: Possible routes by which vulnerable neuronal types may be subject to neuroinvasion by an unknown pathogen. J Neural Transm (Vienna). 2003;110:517–536. 12721813 10.1007/s00702-002-0808-2

[3] Surmeier DJ , Obeso JA, Halliday GM. Selective neuronal vulnerability in Parkinson disease. Nat Rev Neurosci. 2017;18:101–113. 28104909 10.1038/nrn.2016.178 PMC5564322

[4] Challis C , Hori A, Sampson TR, et al Gut-seeded α-synuclein fibrils promote gut dysfunction and brain pathology specifically in aged mice. Nat Neurosci. 2020;23:327–336. 32066981 10.1038/s41593-020-0589-7 PMC7065967

[5] Recasens A , Dehay B, Bové J, et al Lewy Body extracts from Parkinson disease brains trigger α-synuclein pathology and neurodegeneration in mice and monkeys. Ann Neurol. 2014;75:351–362. 24243558 10.1002/ana.24066

[6] Borghammer P , Van Den Berge N. Brain-first versus gut-first Parkinson's disease: A hypothesis. J Parkinsons Dis. 2019;9(s2):S281–s295. 31498132 10.3233/JPD-191721 PMC6839496

[7] Prusiner SB , Woerman AL, Mordes DA, et al Evidence for α-synuclein prions causing multiple system atrophy in humans with parkinsonism. Proc Natl Acad Sci U S A. 2015;112:E5308–E5317. 26324905 10.1073/pnas.1514475112 PMC4586853

[8] Polinski NK . A summary of phenotypes observed in the in vivo rodent alpha-synuclein preformed fibril model. J Parkinsons Dis. 2021;11:1555–1567. 34486988 10.3233/JPD-212847 PMC8609716

[9] Mahul-Mellier A-L , Burtscher J, Maharjan N, et al The process of Lewy body formation, rather than simply α-synuclein fibrillization, is one of the major drivers of neurodegeneration. Proc Natl Acad Sci U S A. 2020;117:4971–4982. 32075919 10.1073/pnas.1913904117 PMC7060668

[10] Volpicelli-Daley LA , Luk KC, Patel TP, et al Exogenous α-synuclein fibrils induce Lewy body pathology leading to synaptic dysfunction and neuron death. Neuron. 2011;72:57–71. 21982369 10.1016/j.neuron.2011.08.033 PMC3204802

[11] Castonguay AM , Gravel C, Lévesque M. Treating Parkinson's disease with antibodies: Previous studies and future directions. J Parkinsons Dis. 2021;11:71–92. 33104039 10.3233/JPD-202221 PMC7990466

[12] Ihse E , Yamakado H, van Wijk XM, Lawrence R, Esko JD, Masliah E. Cellular internalization of alpha-synuclein aggregates by cell surface heparan sulfate depends on aggregate conformation and cell type. Sci Rep. 2017;7:9008. 28827536 10.1038/s41598-017-08720-5 PMC5566500

[13] Scheiblich H , Dansokho C, Mercan D, et al Microglia jointly degrade fibrillar alpha-synuclein cargo by distribution through tunneling nanotubes. Cell Sep. 2021;184:5089–5106.e21. 10.1016/j.cell.2021.09.007 PMC8527836 34555357

[14] Rostami J , Mothes T, Kolahdouzan M, et al Crosstalk between astrocytes and microglia results in increased degradation of α-synuclein and amyloid-β aggregates. J Neuroinflammation. 2021;18:124. 34082772 10.1186/s12974-021-02158-3 PMC8173980

[15] Lee H-J , Suk J-E, Bae E-J, Lee S-J. Clearance and deposition of extracellular α-synuclein aggregates in microglia. Biochem Biophys Res Commun. 2008;372:423–428. 18492487 10.1016/j.bbrc.2008.05.045

[16] Long H , Zhang S, Zeng S, et al Interaction of RAGE with α-synuclein fibrils mediates inflammatory response of microglia. Cell Rep. 2022;40:111401. 36130498 10.1016/j.celrep.2022.111401

[17] Fellner L , Irschick R, Schanda K, et al Toll-like receptor 4 is required for α-synuclein dependent activation of microglia and astroglia. Glia. 2013;61:349–360. 23108585 10.1002/glia.22437 PMC3568908

[18] Kim C , Ho D-H, Suk J-E, et al Neuron-released oligomeric α-synuclein is an endogenous agonist of TLR2 for paracrine activation of microglia. Nat Commun. 2013;4:1562. 23463005 10.1038/ncomms2534 PMC4089961

[19] Panicker N , Sarkar S, Harischandra DS, et al Fyn kinase regulates misfolded α-synuclein uptake and NLRP3 inflammasome activation in microglia. J Exp Med. 2019;216:1411–1430. 31036561 10.1084/jem.20182191 PMC6547864

[20] Scheiblich H , Bousset L, Schwartz S, et al Microglial NLRP3 inflammasome activation upon TLR2 and TLR5 ligation by distinct α-synuclein assemblies. J Immunol. 2021;207:2143–2154. 34507948 10.4049/jimmunol.2100035 PMC8490941

[21] Healy LM , Yaqubi M, Ludwin S, Antel J. Species differences in immune-mediated CNS tissue injury and repair: A (neuro)inflammatory topic. Glia. 2020;68:811–829. 31724770 10.1002/glia.23746

[22] Geirsdottir L , David E, Keren-Shaul H, et al Cross-species single-cell analysis reveals divergence of the primate microglia program. Cell. 2019;179:1609–1622.e16. 31835035 10.1016/j.cell.2019.11.010

[23] Gosselin D , Skola D, Coufal NG, et al An environment-dependent transcriptional network specifies human microglia identity. Science. 2017;356:eaal3222. 28546318 10.1126/science.aal3222 PMC5858585

[24] Owen DR , Narayan N, Wells L, et al Pro-inflammatory activation of primary microglia and macrophages increases 18 kDa translocator protein expression in rodents but not humans. J Cereb Blood Flow Metab. 2017;37:2679–2690. 28530125 10.1177/0271678X17710182 PMC5536262

[25] Dorion M-F , Yaqubi M, Murdoch HJ, et al Systematic comparison of culture media uncovers phenotypic shift of primary human microglia defined by reduced reliance to CSF1R signaling. Glia. 2023;71:1278–1293. 36680780 10.1002/glia.24338

[26] Healy LM , Perron G, Won S-Y, et al MerTK is a functional regulator of myelin phagocytosis by human myeloid cells. J Immunol. 2016;196:3375–3384. 26962228 10.4049/jimmunol.1502562

[27] Scott RS , McMahon EJ, Pop SM, et al Phagocytosis and clearance of apoptotic cells is mediated by MER. Nature. 2001;411:207–211. 11346799 10.1038/35075603

[28] Chung WS , Clarke LE, Wang GX, et al Astrocytes mediate synapse elimination through MEGF10 and MERTK pathways. Nature. 2013;504:394–400. 24270812 10.1038/nature12776 PMC3969024

[29] D'Cruz PM , Yasumura D, Weir J, et al Mutation of the receptor tyrosine kinase gene Mertk in the retinal dystrophic RCS rat. Hum Mol Genet. 2000;9:645–651. 10699188 10.1093/hmg/9.4.645

[30] Feng W , Yasumura D, Matthes MT, LaVail MM, Vollrath D. Mertk triggers uptake of photoreceptor outer segments during phagocytosis by cultured retinal pigment epithelial cells. J Biol Chem. 2002;277:17016–17022. 11861639 10.1074/jbc.M107876200

[31] Lemke G . Biology of the TAM receptors. Cold Spring Harb Perspect Biol. 2013;5:a009076. 24186067 10.1101/cshperspect.a009076 PMC3809585

[32] Rothlin CV , Carrera-Silva EA, Bosurgi L, Ghosh S. TAM receptor signaling in immune homeostasis. Annu Rev Immunol. 2015;33:355–391. 25594431 10.1146/annurev-immunol-032414-112103 PMC4491918

[33] Butovsky O , Jedrychowski MP, Moore CS, et al Identification of a unique TGF-β-dependent molecular and functional signature in microglia. Nat Neurosci. 2014;17:131–143. 24316888 10.1038/nn.3599 PMC4066672

[34] Durafourt BA , Moore CS, Blain M, Antel JP. Isolating, culturing, and polarizing primary human adult and fetal microglia. Methods Mol Biol. 2013;1041:199–211. 23813381 10.1007/978-1-62703-520-0_19

[35] Chen CX , Abdian N, Maussion G, et al A multistep workflow to evaluate newly generated iPSCs and their ability to generate different cell types. Methods Protoc. 2021;4:50. 34287353 10.3390/mps4030050 PMC8293472

[36] McQuade A , Coburn M, Tu CH, Hasselmann J, Davtyan H, Blurton-Jones M. Development and validation of a simplified method to generate human microglia from pluripotent stem cells. Mol Neurodegener. 2018;13:67. 30577865 10.1186/s13024-018-0297-x PMC6303871

[37] Bourgey M , Dali R, Eveleigh R, et al Genpipes: An open-source framework for distributed and scalable genomic analyses. Gigascience. 2019;8:giz037. 31185495 10.1093/gigascience/giz037 PMC6559338

[38] Sasaki A , Arawaka S, Sato H, Kato T. Sensitive western blotting for detection of endogenous Ser129-phosphorylated α-synuclein in intracellular and extracellular spaces. Sci Rep. 2015;5:14211. 26381815 10.1038/srep14211 PMC4585644

[39] Maneca D-L , Luo W, Krahn A, et al Production of recombinant α synuclein monomers and preformed fibrils (PFFs) (V3.0). Zenodo. 2022; 10.5281/zenodo.6430401

[40] Del Cid Pellitero E , Shlaifer R, Luo W, et al Quality control characterization of α-synuclein preformed fibrils (PFFs) (V3.0). Zenodo. 2019; 10.5281/zenodo.6524616

[41] Healy LM , Jang JH, Won S-Y, et al MerTK-mediated regulation of myelin phagocytosis by macrophages generated from patients with MS. Neurol Neuroimmunol Neuroinflamm. 2017;4:e402. 29379818 10.1212/NXI.0000000000000402 PMC5777663

[42] Hughes AJ , Daniel SE, Kilford L, Lees AJ. Accuracy of clinical diagnosis of idiopathic Parkinson's disease: A clinico-pathological study of 100 cases. J Neurol Neurosurg Psychiatry. 1992;55:181–184. 1564476 10.1136/jnnp.55.3.181 PMC1014720

[43] Postuma RB , Berg D, Stern M, et al MDS Clinical diagnostic criteria for Parkinson's disease. Mov Disord. 2015;30:1591–1601. 26474316 10.1002/mds.26424

[44] Carson AR , Smith EN, Matsui H, et al Effective filtering strategies to improve data quality from population-based whole exome sequencing studies. BMC Bioinformatics. 2014;15:125. 24884706 10.1186/1471-2105-15-125 PMC4098776

[45] Zhao Z , Bi W, Zhou W, VandeHaar P, Fritsche LG, Lee S. UK Biobank whole-exome sequence binary phenome analysis with robust region-based rare-variant test. Am J Hum Genet. 2020;106:3–12. 31866045 10.1016/j.ajhg.2019.11.012 PMC7042481

[46] Lee S , Teslovich TM, Boehnke M, Lin X. General framework for meta-analysis of rare variants in sequencing association studies. Am J Hum Genet. 2013;93:42–53. 23768515 10.1016/j.ajhg.2013.05.010 PMC3710762

[47] Kamath T , Abdulraouf A, Burris SJ, et al Single-cell genomic profiling of human dopamine neurons identifies a population that selectively degenerates in Parkinson's disease. Nat Neurosci. 2022;25:588–595. 35513515 10.1038/s41593-022-01061-1 PMC9076534

[48] Miksa M , Komura H, Wu R, Shah KG, Wang P. A novel method to determine the engulfment of apoptotic cells by macrophages using pHrodo succinimidyl ester. J Immunol Methods. 2009;342(1–2):71–77. 19135446 10.1016/j.jim.2008.11.019 PMC2675277

[49] Söderberg O , Gullberg M, Jarvius M, et al Direct observation of individual endogenous protein complexes in situ by proximity ligation. Nat Methods. 2006;3:995–1000. 17072308 10.1038/nmeth947

[50] Zhang W , DeRyckere D, Hunter D, et al UNC2025, a potent and orally bioavailable MER/FLT3 dual inhibitor. J Med Chem. 2014;57:7031–7041. 25068800 10.1021/jm500749d PMC4148167

[51] Junker F , Gordon J, Qureshi O. Fc gamma receptors and their role in antigen uptake, presentation, and T cell activation. Review. Front Immunol. 2020;11:1393. 32719679 10.3389/fimmu.2020.01393 PMC7350606

[52] Henriksen L , Grandal MV, Knudsen SL, van Deurs B, Grøvdal LM. Internalization mechanisms of the epidermal growth factor receptor after activation with different ligands. PLoS One. 2013;8:e58148. 23472148 10.1371/journal.pone.0058148 PMC3589378

[53] Lemke G , Rothlin CV. Immunobiology of the TAM receptors. Nat Rev Immunol. 2008;8:327–336. 18421305 10.1038/nri2303 PMC2856445

[54] Landeck N , Hall H, Ardah MT, et al A novel multiplex assay for simultaneous quantification of total and S129 phosphorylated human alpha-synuclein. Mol Neurodegener. 2016;11:61. 27549140 10.1186/s13024-016-0125-0 PMC4994244

[55] Moors TE , Mona D, Luehe S, et al Multi-platform quantitation of alpha-synuclein human brain proteoforms suggests disease-specific biochemical profiles of synucleinopathies. Acta Neuropathol Commun. 2022;10:82. 35659116 10.1186/s40478-022-01382-z PMC9164351

[56] Underhill DM , Goodridge HS. Information processing during phagocytosis. Nat Rev Immunol. 2012;12:492–502. 22699831 10.1038/nri3244 PMC5570470

[57] Li N , Stewart T, Sheng L, et al Immunoregulation of microglial polarization: An unrecognized physiological function of α-synuclein. J Neuroinflammation. 2020;17:272. 32943057 10.1186/s12974-020-01940-z PMC7500012

[58] Trudler D , Nazor KL, Eisele YS, et al Soluble α-synuclein–antibody complexes activate the NLRP3 inflammasome in hiPSC-derived microglia. Proc Natl Acad Sci U S A. 2021;118:e2025847118. 10.1073/pnas.2025847118 PMC8054017 33833060

[59] Pike AF , Varanita T, Herrebout MAC, et al α-Synuclein evokes NLRP3 inflammasome-mediated IL-1β secretion from primary human microglia. Glia. 2021;69:1413–1428. 33506583 10.1002/glia.23970 PMC8247862

[60] Abdelmotilib H , Maltbie T, Delic V, et al α-Synuclein fibril-induced inclusion spread in rats and mice correlates with dopaminergic neurodegeneration. Neurobiol Dis. 2017;105:84–98. 28576704 10.1016/j.nbd.2017.05.014 PMC5701756

[61] Tarutani A , Suzuki G, Shimozawa A, et al The effect of fragmented pathogenic α-synuclein seeds on prion-like propagation. J Biol Chem. 2016;291:18675–18688. 27382062 10.1074/jbc.M116.734707 PMC5009244

[62] Lew ED , Oh J, Burrola PG, et al Differential TAM receptor-ligand-phospholipid interactions delimit differential TAM bioactivities. Elife. 2014;3:e03385. 25265470 10.7554/eLife.03385 PMC4206827

[63] Burstyn-Cohen T , Lew ED, Través PG, Burrola PG, Hash JC, Lemke G. Genetic dissection of TAM receptor-ligand interaction in retinal pigment epithelial cell phagocytosis. Neuron. 2012;76:1123–1132. 23259948 10.1016/j.neuron.2012.10.015 PMC3530147

[64] Duncan JL , LaVail MM, Yasumura D, et al An RCS-like retinal dystrophy phenotype in mer knockout mice. Invest Ophthalmol Vis Sci. 2003;44:826–838. 12556419 10.1167/iovs.02-0438

[65] Lu Q , Gore M, Zhang Q, et al Tyro-3 family receptors are essential regulators of mammalian spermatogenesis. Nature. 1999;398:723–728. 10227296 10.1038/19554

[66] Zagórska A , Través PG, Lew ED, Dransfield I, Lemke G. Diversification of TAM receptor tyrosine kinase function. Nat Immunol. 2014;15:920–928. 25194421 10.1038/ni.2986 PMC4169336

[67] Wanke F , Gutbier S, Rümmelin A, et al Ligand-dependent kinase activity of MERTK drives efferocytosis in human iPSC-derived macrophages. Cell Death Dis. 2021;12:538. 34035216 10.1038/s41419-021-03770-0 PMC8149813

[68] Stevenson TJ , Murray HC, Turner C, Faull RLM, Dieriks BV, Curtis MA. α-Synuclein inclusions are abundant in non-neuronal cells in the anterior olfactory nucleus of the Parkinson’s disease olfactory bulb. Sci Rep. 2020;10:6682. 32317654 10.1038/s41598-020-63412-x PMC7174302

[69] Emmanouilidou E , Elenis D, Papasilekas T, et al Assessment of α-synuclein secretion in mouse and human brain parenchyma. PLoS One. 2011;6:e22225. 21779395 10.1371/journal.pone.0022225 PMC3136497

[70] Yamada K , Iwatsubo T. Extracellular α-synuclein levels are regulated by neuronal activity. Mol Neurodegener. 2018;13:9. 29467003 10.1186/s13024-018-0241-0 PMC5822605

[71] Park J , Choi Y, Jung E, Lee SH, Sohn JW, Chung WS. Microglial MERTK eliminates phosphatidylserine-displaying inhibitory post-synapses. EMBO J. 2021;40:e107121. 34013588 10.15252/embj.2020107121 PMC8327958

[72] Damisah EC , Hill RA, Rai A, et al Astrocytes and microglia play orchestrated roles and respect phagocytic territories during neuronal corpse removal in vivo. Sci Adv. 2020;6:eaba3239. 32637606 10.1126/sciadv.aba3239 PMC7319765

[73] Huang Y , Happonen KE, Burrola PG, et al Microglia use TAM receptors to detect and engulf amyloid β plaques. Nat Immunol. 2021;22:586–594. 33859405 10.1038/s41590-021-00913-5 PMC8102389

[74] Huang Y , Lemke G. Early death in a mouse model of Alzheimer’s disease exacerbated by microglial loss of TAM receptor signaling. Proc Natl Acad Sci U S A. 2022;119:e2204306119. 10.1073/pnas.2204306119 PMC9564325 36191221

[75] Pereira JB , Janelidze S, Strandberg O, et al Microglial activation protects against accumulation of tau aggregates in nondemented individuals with underlying Alzheimer’s disease pathology. Nat Aging. 2022;2:1138–1144. 37118533 10.1038/s43587-022-00310-z PMC10154192

[76] Brosseron F , Maass A, Kleineidam L, et al Soluble TAM receptors sAXL and sTyro3 predict structural and functional protection in Alzheimer's disease. Neuron. 2022;110:1009–1022.e4. 34995486 10.1016/j.neuron.2021.12.016

[77] Brown GC , Neher JJ. Eaten alive! cell death by primary phagocytosis: ‘phagoptosis’. Trends Biochem Sci. 2012;37:325–332. 22682109 10.1016/j.tibs.2012.05.002

[78] Kedage V , Ellerman D, Chen Y, et al Harnessing MerTK agonism for targeted therapeutics. MAbs. 2020;12:1685832. 31852344 10.1080/19420862.2019.1685832 PMC6927767

[79] Samentar LP , Salazar A, Pan P-P, et al A novel hybrid protein promotes Aβ clearance and reduces inflammatory response through MerTK. bioRxiv. [Preprint] doi:10.1101/2021.11.03.467048

