# Supplementary material for: MerTK is a mediator of alpha-synuclein fibril uptake by human microglia
Source: Brain. 2023 Sep 6;147(2):427–43. doi: 10.1093/brain/awad298 (PMC10834256; doi:10.1093/brain/awad298)
Supplement: awad298_Supplementary_Data [file awad298_supplementary_data.zip › brain-2023-00469-File009.pdf]

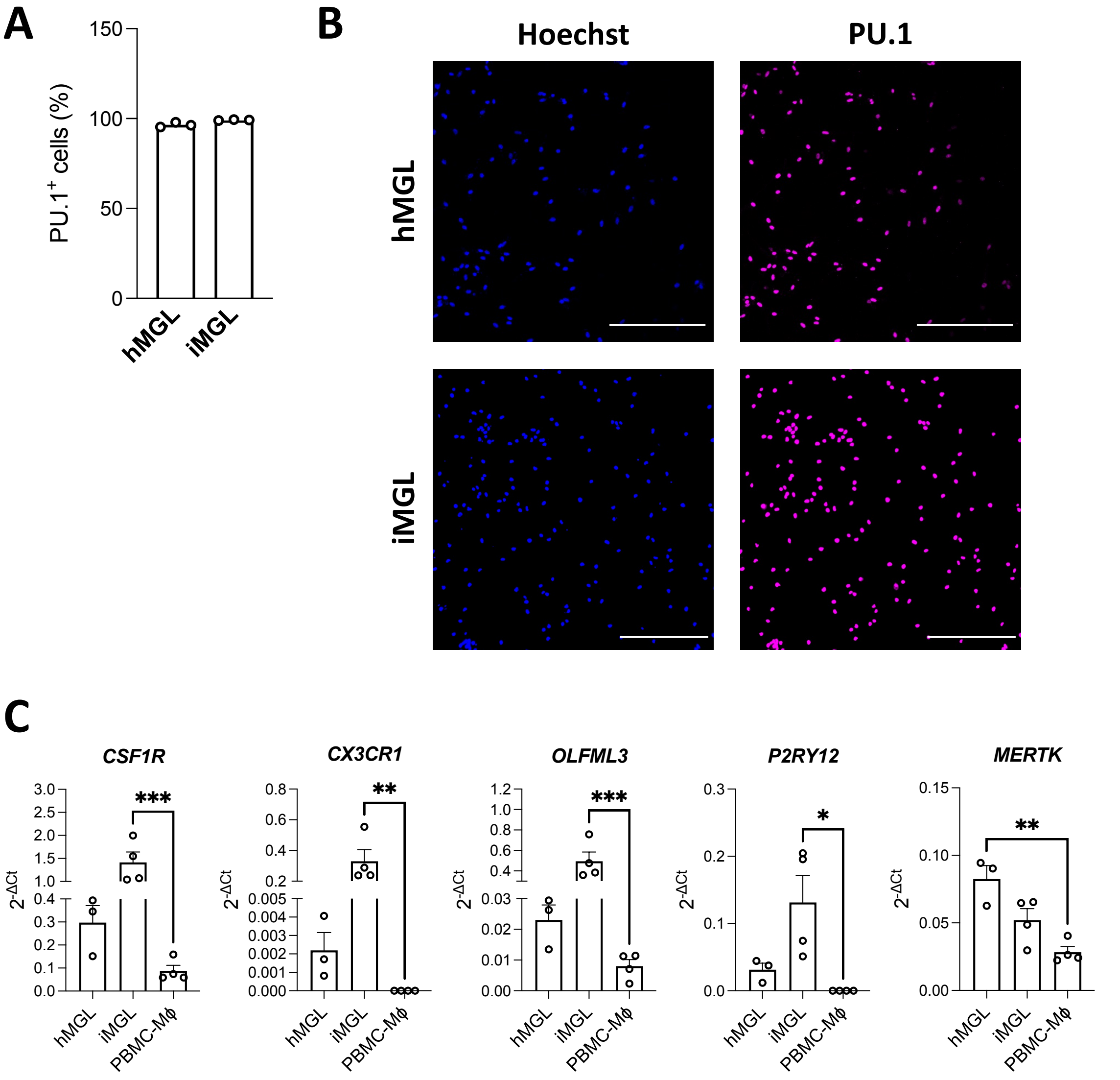

Supplementary Figure 1. hMGL and iMGL characterization. Quantification of PU.1+ cells (A) and representative immunostaining (B) of hMGL and iMGL. Mean +/- SEM of n = 3. (C) qRT-PCR assessment of microglia markers in hMGL, iMGL and PBMC-Mϕ. One-way ANOVA were performed, followed by Dunnett's post hoc tests, mean +/- SEM of n = 3 for hMGL, 4 for iMGL, and 4 for PBMC-Mϕ, $*p < 0.05$, $**p < 0.01$, $***p < 0.001$ vs PBMC-Mϕ.

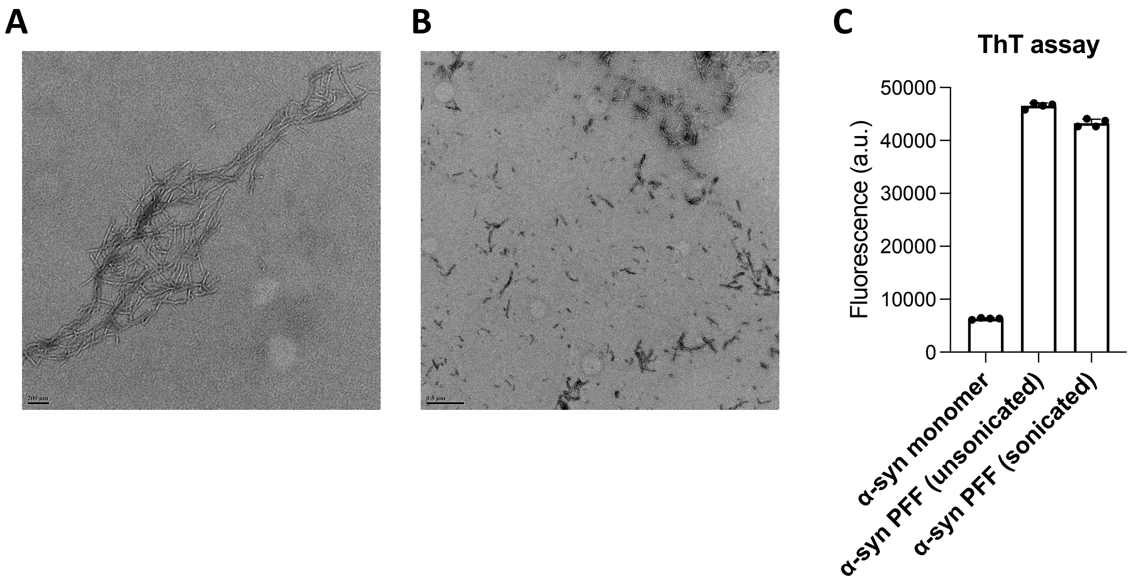

Supplementary Figure 2. Quality control of α-syn PFFs. Representative electron microscopy images of unsonicated (A) and sonicated (B) α-syn PFFs. Scale bar = 200 nm in (A) and 500 nm in (B). (C) Representative thioflavin T (ThT) assay. α-syn monomers or PFFs were mixed with ThT and incubated for 20 minutes, following which fluorescent signals were read by a microplate reader with excitation at 450 nm and emission at 490 nm. PBS was used for background subtraction. Mean +/- standard deviation of four technical replicates are presented, a.u. = arbitrary unit.

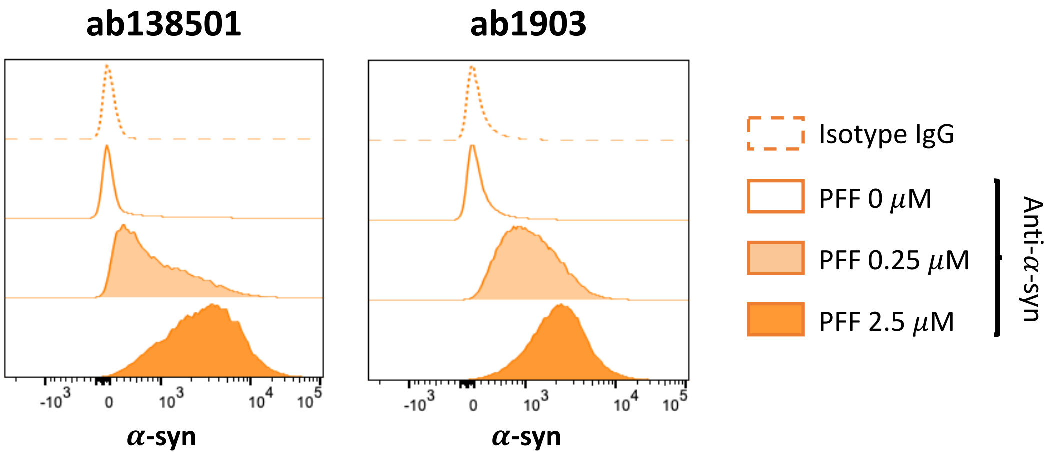

Supplementary Figure 3. Specificity of anti-α-syn antibodies. Flow cytometry staining of α-syn on iMGL treated of not with α-syn PFFs for 30 minutes on ice.

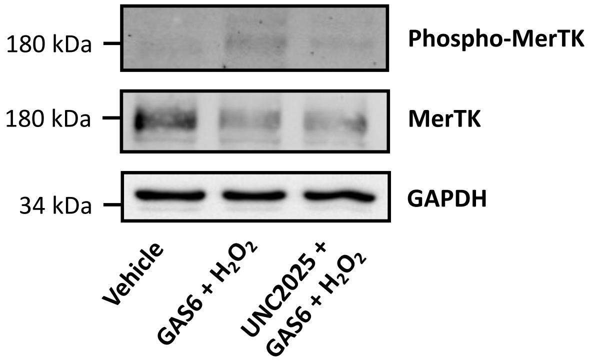

Supplementary Figure 4. Inhibitory effect of UNC2025 on MerTK activation. iMGL were pretreated or not with UNC2025 for one hour and then treated with vehicle or GAS6 (400 ng/mL) and hydrogen peroxide (H_2O_2; 4 mM) for 10 minutes. Western blotting of phosphorylated MerTK, total MerTK and GAPDH was carried out.

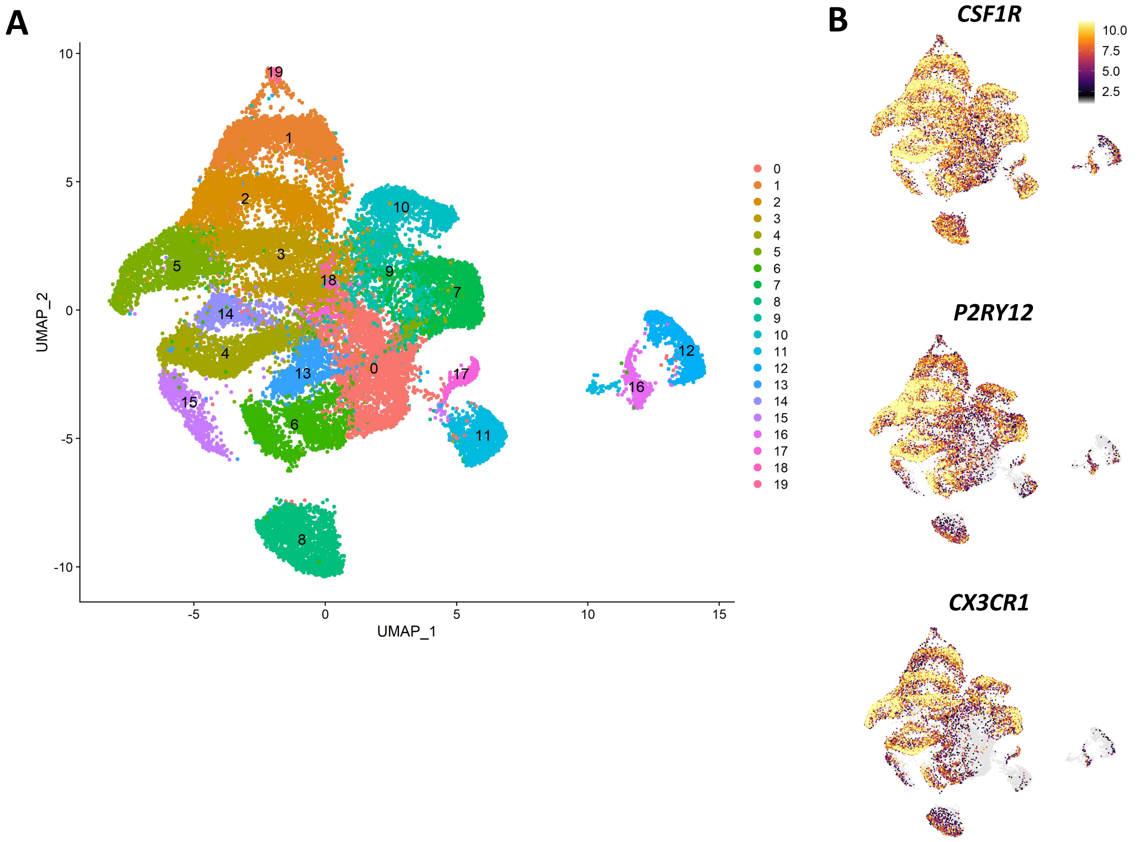

Supplementary Figure 5. Microglia clusters identified by snRNAseq. (A) uniform manifold approximation and projection (UMAP) of microglia clusters identified in the snRNAseq data from Kamath *et al.*, 2022. (B) Expression of microglia canonical markers in the identified clusters. Color scale represents gene expression level.

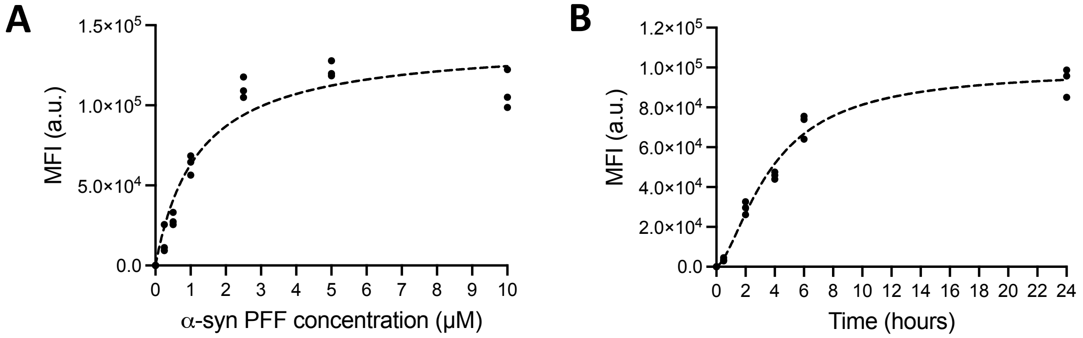

Supplementary Figure 6. α-syn PFF internalization by iMGL in function of α-syn PFF concentration and time. (A) Quantification of mean green fluorescence intensity (MFI) in iMGL challenged with pHrodo™ Green-labelled α-syn PFFs for two hours. Data from three technical replicates are presented. a.u. = arbitrary unit. Dotted line represents fitted nonlinear regression curve. (B) MFI in iMGL challenged with 1 μM pHrodo™ Green-labelled α-syn PFFs for varying lengths of time. Data from three technical replicates are presented. a.u. = arbitrary unit. Dotted line represents fitted nonlinear regression curve.

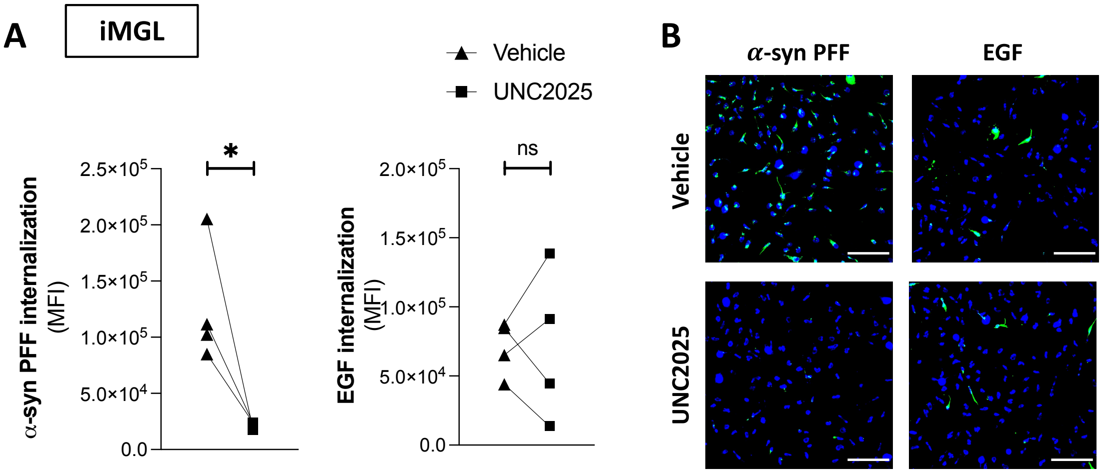

Supplementary Figure 7. Effect of UNC2025 on microglial uptake of Alexa Fluor 488-labelled α-syn PFFs. iMGL were pretreated with vehicle or UNC2025 (3 µM) for one hour and then challenged with Alexa Fluor 488-labelled α-syn PFFs or EGF for two hours. (A) Quantification of mean green fluorescence intensity (MFI). Paired t-tests were performed. Mean of $n = 4$, *$p < 0.05$, ns = non-significant. (B) Representative fluorescence images of iMGL counterstained with Hoechst 33342 (blue). Scale bar = 150 μm.

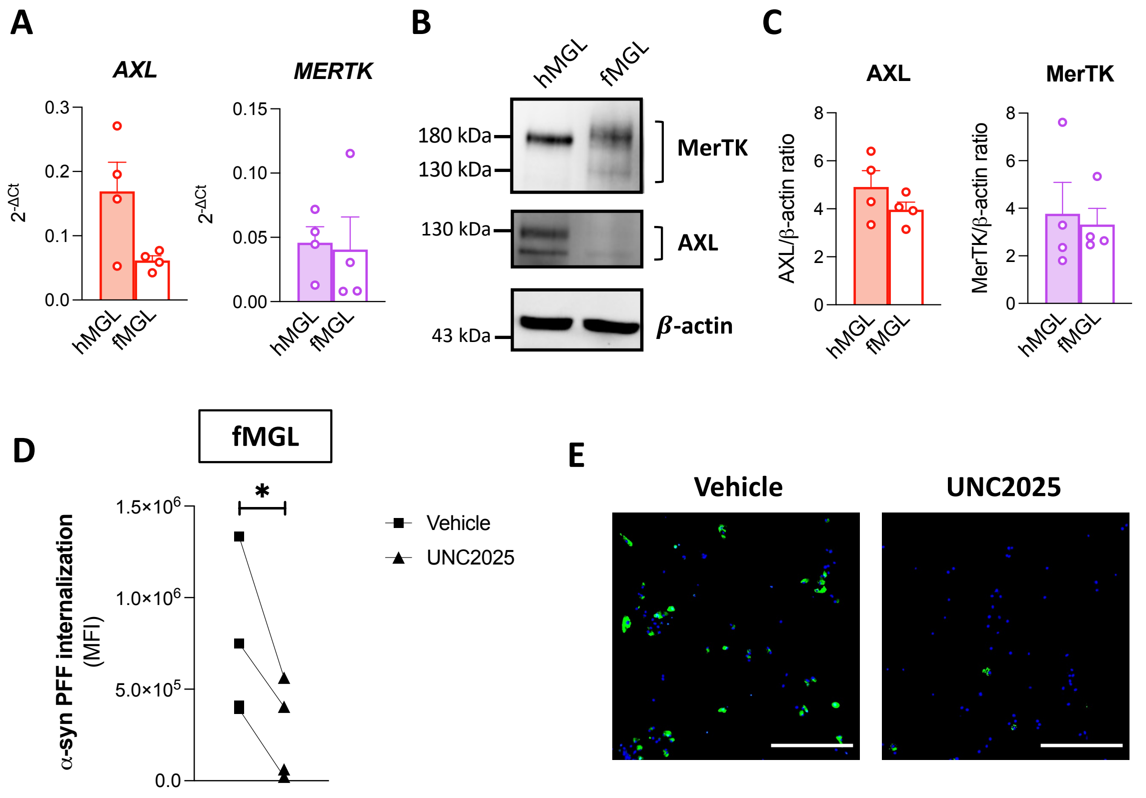

Supplementary Figure 8. Effect of UNC2025 on fMGL uptake of α-syn PFF. (A) qRT-PCR assessment of *AXL* and *MERTK* expression in hMGL and fMGL. Mann-Whitney tests were performed. Mean +/- SEM of $n = 4$. (B) Western blot of AXL, MerTK and β-actin. (C) Quantification of AXL and MerTK expression using β-actin as a loading control. T-tests were performed. Mean +/- SEM of $n = 4$. (D-E) fMGL were pretreated with vehicle or UNC2025 (3 µM) for one hour and then challenged with pHrodo^{TM} Green-labelled α-syn PFFs for two hours. (D) Mean green fluorescence intensity (MFI) was measured. A paired t-test was performed, mean of $n = 4$, $*p < 0.05$. (E) Representative images of fMGL counterstained with Hoechst 33342 (blue). Scale bar = 200 μm.

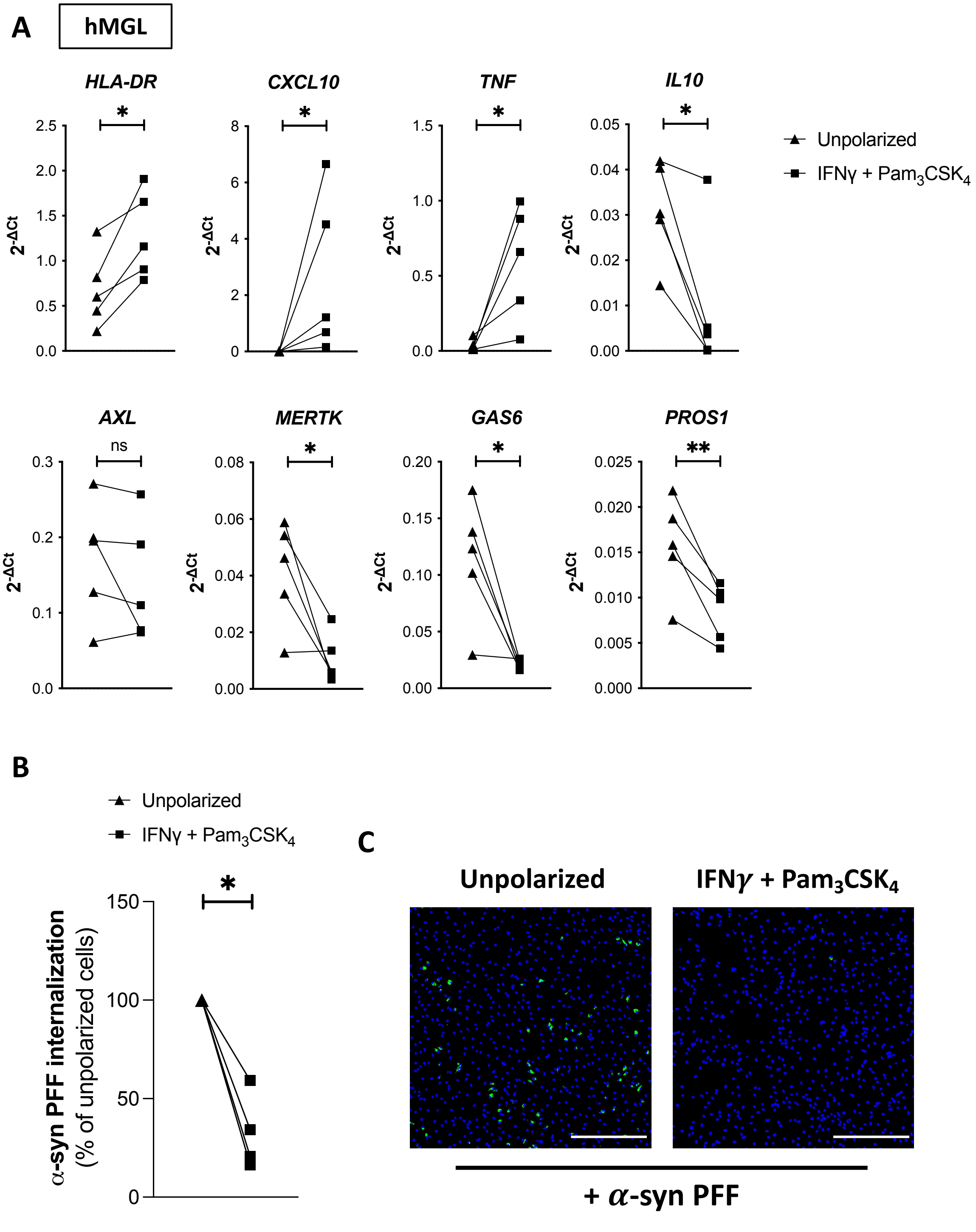

Supplementary Figure 9. Effect of inflammatory polarization on hMGL ability to internalize α-syn PFFs. hMGL were polarized using IFNγ (20 ng/mL) and Pam_3CSK_4 (100 ng/mL) treatment for 48 hours or left unpolarized. (A) qRT-PCR data. Paired t-tests were performed. Mean of $n = 5$, ns = non-significant, $*p < 0.05$, $**p < 0.01$. (B)

Quantification of mean green fluorescence intensity (MFI) per cell in hMGL culture challenged with pHrodo^{TM} Green-labelled α-syn PFFs for two hours. Data were normalized to the unpolarized conditions. A paired t-test was performed. Mean of $n = 4$, $*p < 0.05$. (C) Representative fluorescence images of cells challenged with pHrodo^{TM} Green-labelled α-syn PFFs for two hours and counterstained with Hoechst 33342 (blue). Scale bar = 300 μm.

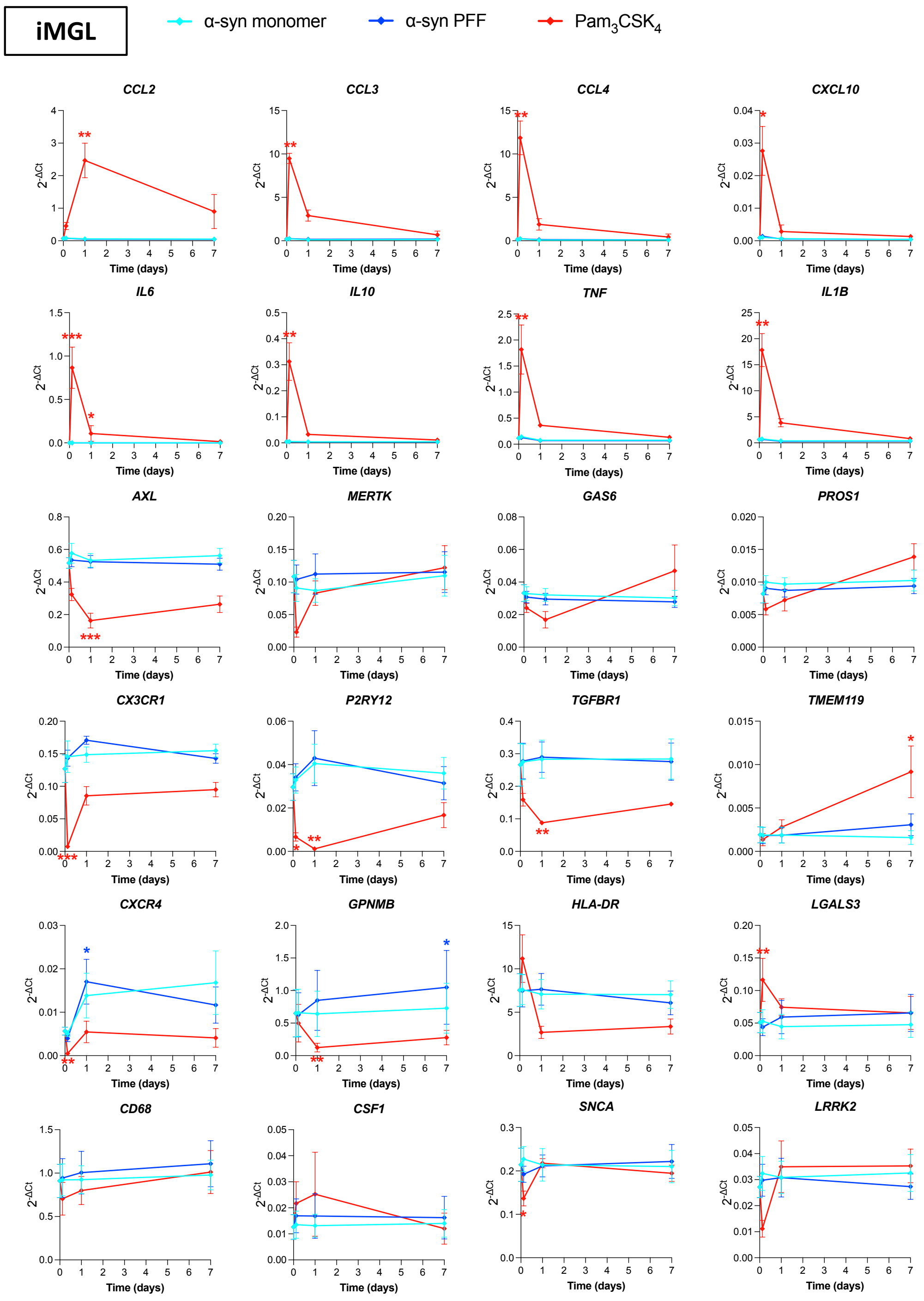

Supplementary Figure 10. Transcriptional changes in microglia following α-syn exposure. iMGL were exposed to a single treatment of α-syn monomers (1 μM), α-syn PFFs (1 μM) or Pam_3CSK_4 (100 ng/mL) for three hours, one day or seven days. Expression

of genes were assessed by qRT-PCR. Friedmann tests were performed, followed by Dunn's post hoc test. Mean +/- SEM of $n = 5$, *$p < 0.05$, **$p < 0.01$, ***$p < 0.001$ vs control.

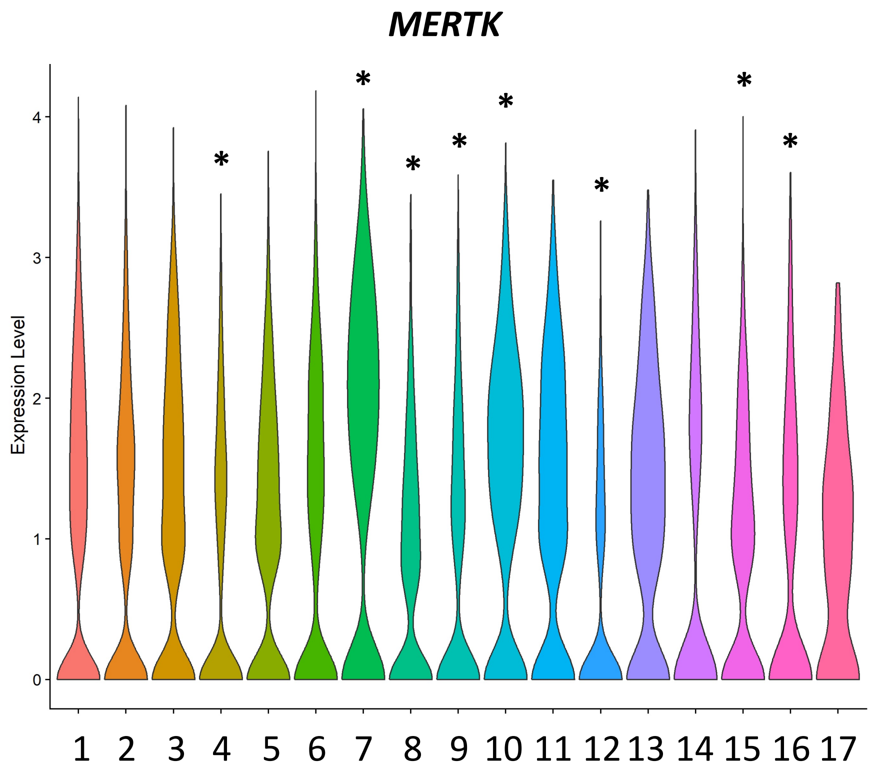

Supplementary Figure 11. Violin plot depicting *MERTK* expression within microglia clusters. Asterix denotes clusters that are significantly enriched ($p < 0.05$) in *MERTK*.

A

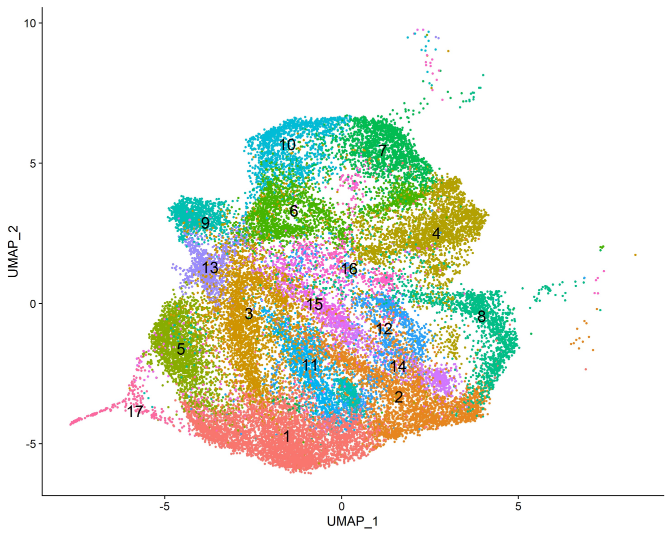

B

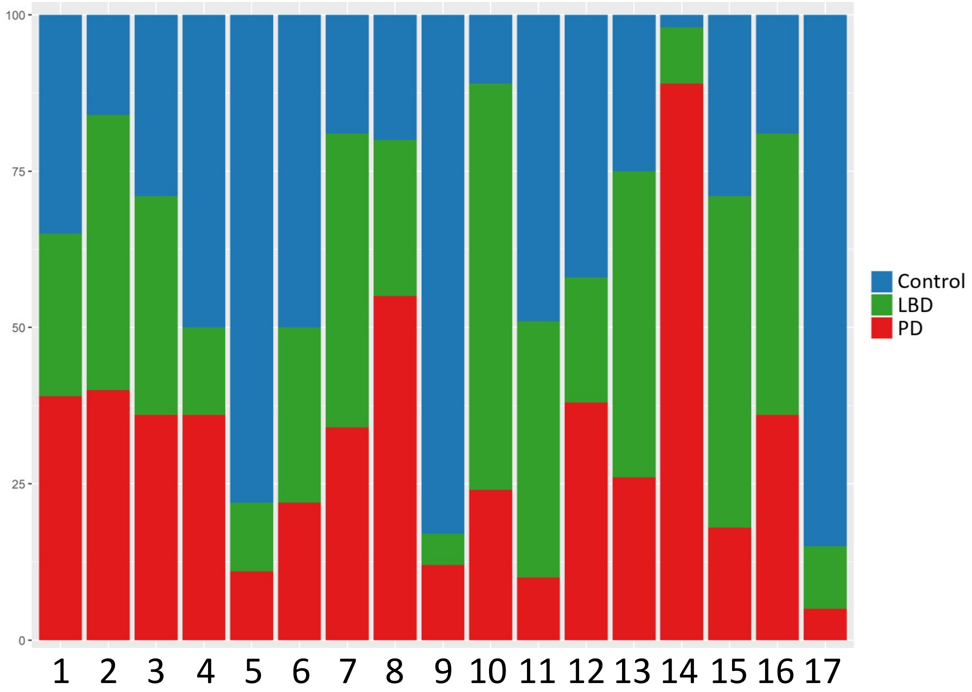

Supplementary Figure 12. Contributions of diagnosis groups to microglia clusters identified by snRNAseq. (A) UMAP plots showing the identified microglia clusters following data integration. (B) Bar graph showing the relative contributions of control (CTL), Lewy body dementia (LBD) and Parkinson's disease (PD) patient-derived microglia to each microglia cluster.

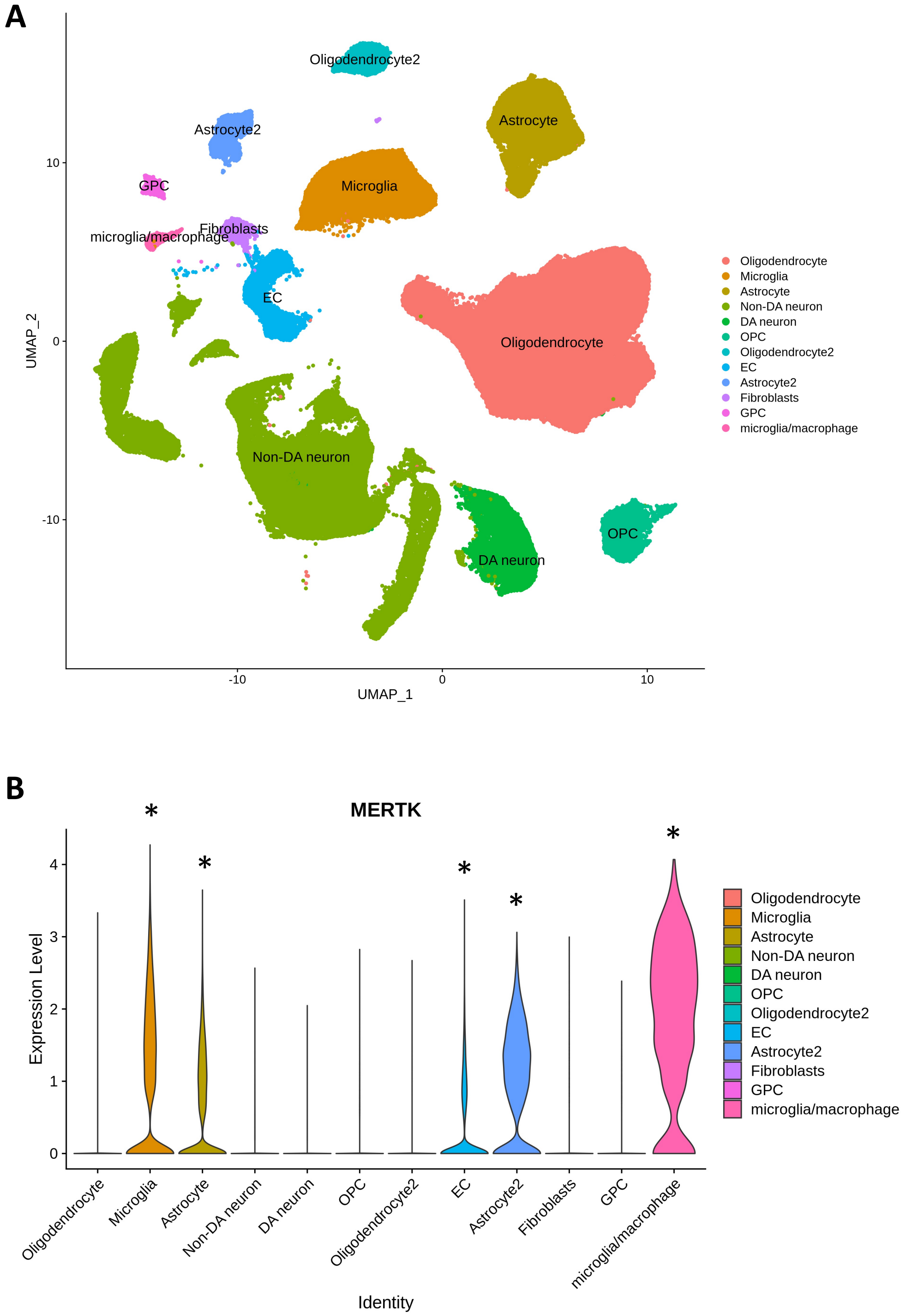

Supplementary Figure 13. *MERTK* expression in human substantia nigra. (A) UMAP showing cell types identified in the snRNAseq dataset from Kamath *et al.,* 2022. (B) Violin plot showing *MERTK* expression across cell types. *Populations that are significantly ($p < 0.05$) enriched in *MERTK* expression. DA = dopaminergic, OPC = oligodendrocyte progenitor cell, EC = endothelial cell, GPC = glial progenitor cell.

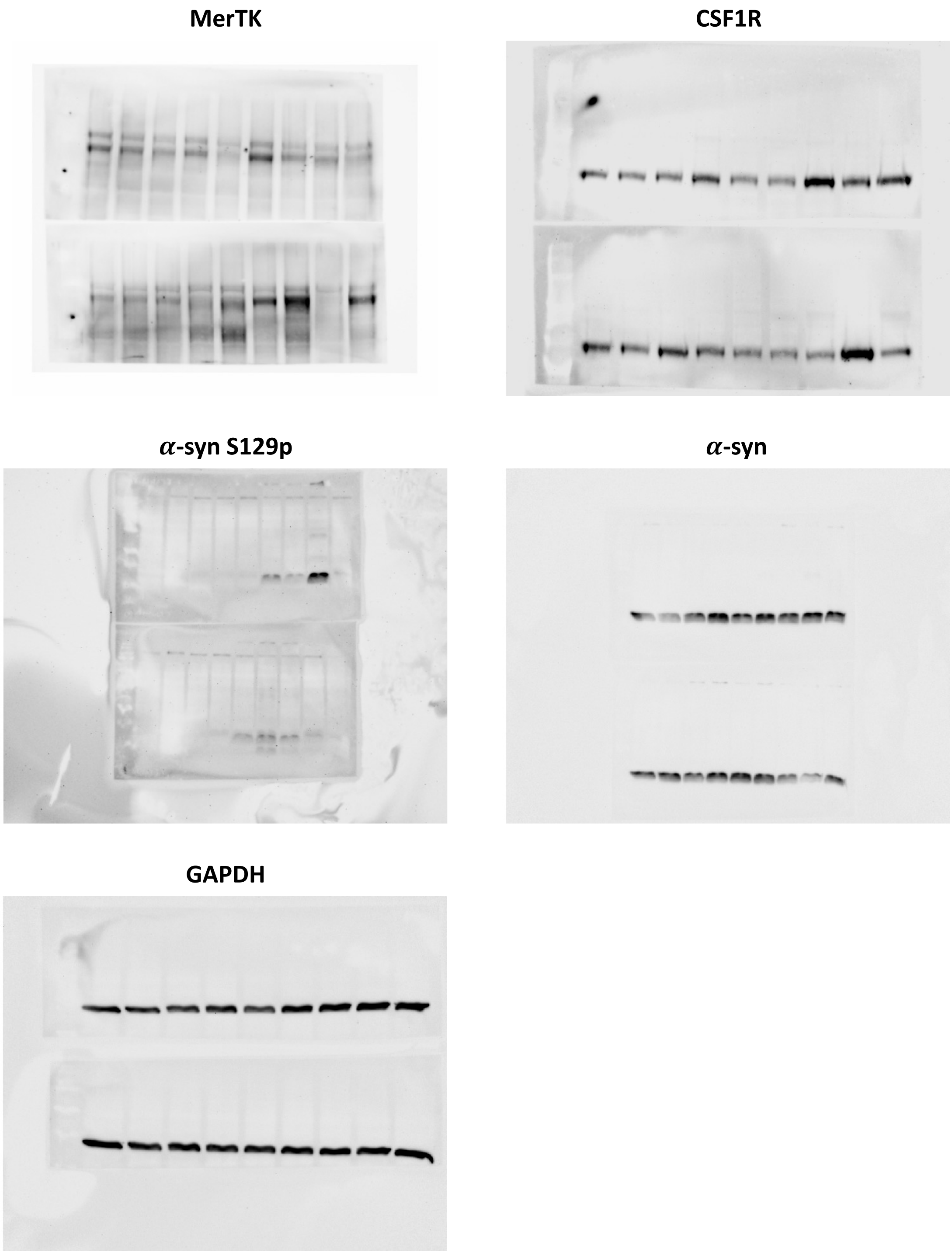

Supplementary Figure 14. Western blots of substantia nigra tissues. Upper membranes lanes 1-5 = control donors, lanes 6-9 = Lewy body dementia patients. Lower membranes lanes 1-4 = control donors, lanes 5-9 = Lewy body dementia patients.

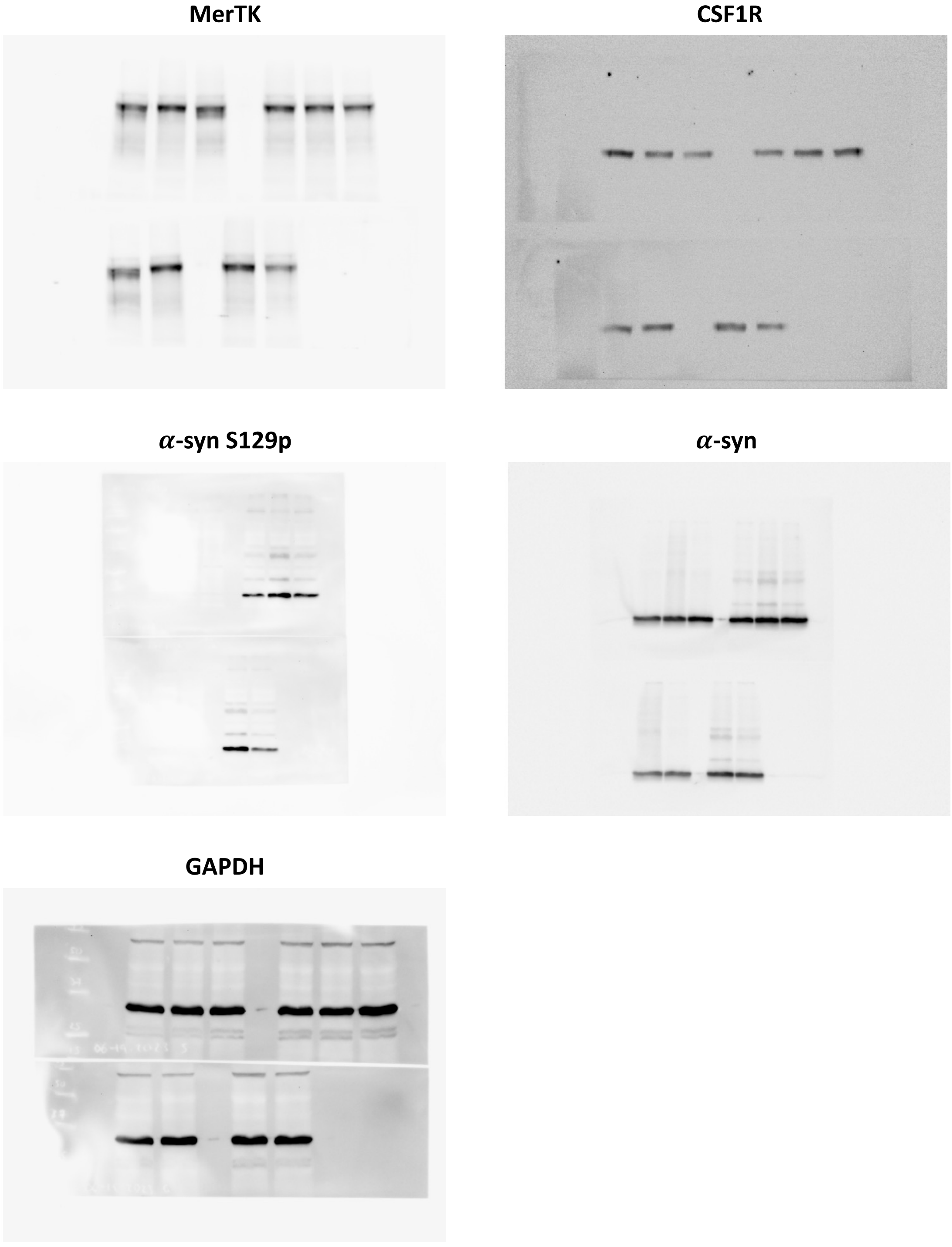

Supplementary Figure 15. Western blots of cortex tissues. Upper membranes lanes 1-3 = control donors, lanes 4-6 = Lewy body dementia patients. Lower membranes lanes 1-2 = control donors, lanes 3-4 = Lewy body dementia patients.
